# Supplementary material for: A syntenic pangenome of Gardnerella reveals novel plasmids and phage, taxonomic boundaries, and species-level stratification of metabolic and virulence potential
Source: Nat Commun. 2026 Jun 20;17:8510. doi: 10.1038/s41467-026-74458-2 (PMC13483204; doi:10.1038/s41467-026-74458-2)
Supplement: Supplementary file 1 [file 41467_2026_74458_MOESM1_ESM.pdf]

Description of Additional Supplementary Files:

Supplementary Data 1: Table of the 677 *Gardnerella* genomes obtained via sequencing of Fredricks and Ravel isolate collections as well as the publicly available genomes from the NCBI database. For each genome, available metadata includes the sample ID, BioSample number, BioProject number, NCBI Assembly Name, Accession and Level, WGS project accession, the biological source for the organism (if known), the country of origin, the sequencing technology, the release data of the NCBI, the number of contigs, the length of the genome, CheckM statistics, the ID of the duplicates Identified by ANI, and whether this genome passed all metrics in to be considered part of the final curated collection.

Supplementary Data 2: Table of the 312 *Gardnerella* genomes as a final curated genome set. This set was generated by the removal of genomes with a genomic duplicate (an average nucleotide identity greater than 99.5%), CheckM contamination (greater than 1%), highly fragmented genomes (greater than 100 contigs), or completeness (less than 90%). Type strains were all retained and exempted from these conditions. Table lists available metadata for each genome, the *cpn60* gene sequence and BLAST results, and taxonomic classification via homology to the Genome Taxonomy Database using GTDB-tk. Final taxonomic classification with the nomenclature proposed in this work is shown for each genome.

Supplementary Data 3: Table shows the 137 genomes from *Bifidobacterium*, *Aeriscardovia*, *Alloscardovia*, *Ancillula*, *Bombiscardovia*, *Galloscardovia*, *Optulatrix*, *Nutricula*, *Pseudoscardovia*, and *Scardovia* used for the comparative analyses in Supplementary Fig. 1. The expanded Alessandri Bifidobacterium groups (Adding Scardovial and Termite Gut groups from within Bifidobacteraceae) and associated hexadecimal (HEX) codes used in the figures are given.

Supplementary Data 4: Table shows the species and subspecies nomenclature proposed for each *Gardnerella* lineage identified in this work, as well as the range of ANI and dDDH values within each group. Included are the unique abbreviations and hexadecimal (HEX) color codes used throughout the figures and tables for these taxa.

Supplementary Data 5: Table shows nodes (gene groups) identified by PPanGGOLiN pangenome analysis of the curated *Gardnerella* genome set. For each node, its product annotation, gene name, partition (persistent, shell, cloud), median length, the number of genomes it was identified in, and its module number (when applicable) are shown. Columns 7-318 show for each genome, the PGAP annotation identifier for the gene grouped into each node.

Supplementary Data 6: Table shows the presence or absence of GapMindpredicted metabolic pathways for the biosynthesis of amino acids and the catabolism of small carbon sources across our curated *Gardnerella* genome set.

Supplementary Data 7: Table shows the number of prophage mapped to either vB_Gva_AB1 or pGamma for each genome in our curated *Gardnerella* genome set as predicted by either PPanGGOLiN synteny and partitioning, VIBRANT, or PhageR. ANI types are assigned based on ANI homology in Supplementary Fig. 5.

Supplementary Data 8: Table shows the presence or absence of PADLOCpredicted genetic defense systems across our curated *Gardnerella* genome set.

Supplementary Data 9: REBASE analysis of methyl-modified motifs detected during PacBio sequencing of *Gardnerella* isolates.

Supplementary Data 10: For the three native *Gardnerella* plasmids identified in our pangenome analysis, table shows plasmid name, length, and nucleotide sequence.

Supplementary Data 11: Table shows the Proksee-predicted open reading frames, their corresponding amino acid sequence, top BLASTp hit, and conserved domain search results for each of the three native *Gardnerella* plasmids.
