# Supplementary material for: A syntenic pangenome of Gardnerella reveals novel plasmids and phage, taxonomic boundaries, and species-level stratification of metabolic and virulence potential
Source: Nat Commun. 2026 Jun 20;17:8510. doi: 10.1038/s41467-026-74458-2 (PMC13483204; doi:10.1038/s41467-026-74458-2)
Supplement: Supplementary file 2 [file 41467_2026_74458_MOESM2_ESM.pdf]

Supplementary Figures

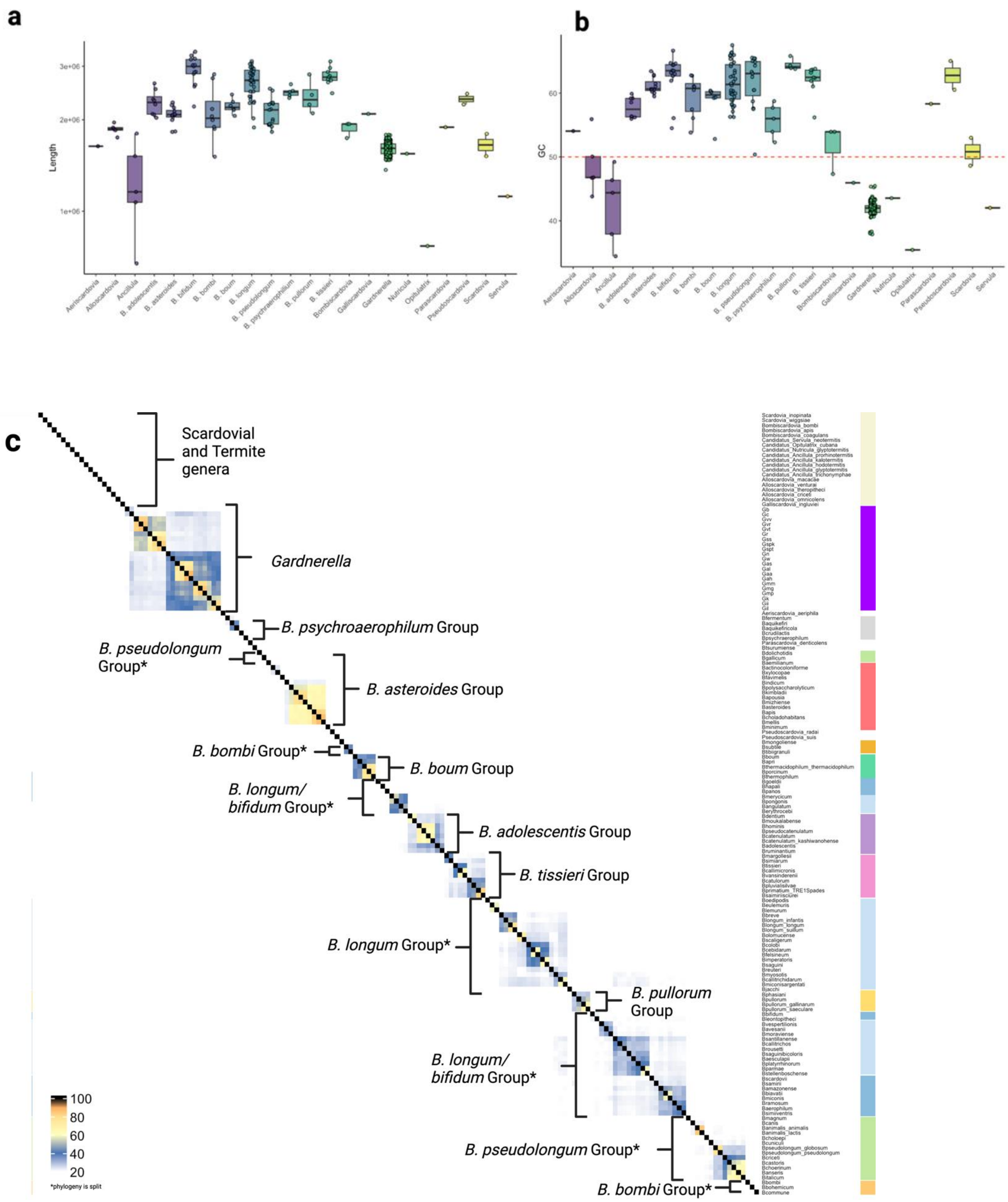

d

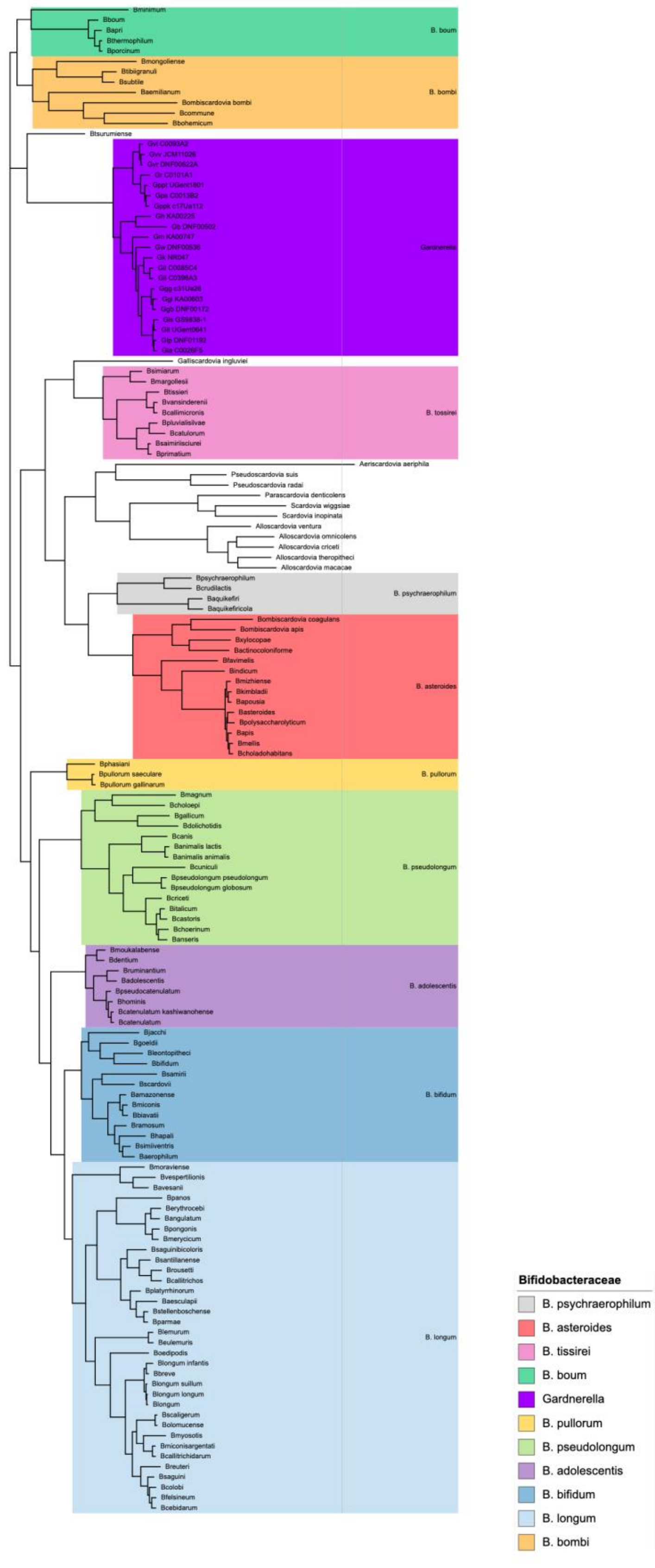
B. boum
B. bombi
Gardnerella
B. tissirei
B. psychraerophilum
B. asteroides
B. pullorum
B. pseudolongum
B. adolescentis
B. bifidum
B. longum
Bifidobacteraceae
B. psychraerophilum
B. asteroides
B. tissirei
B. boum
Gardnerella
B. pullorum
B. pseudolongum
B. adolescentis
B. bifidum
B. longum
B. bombi

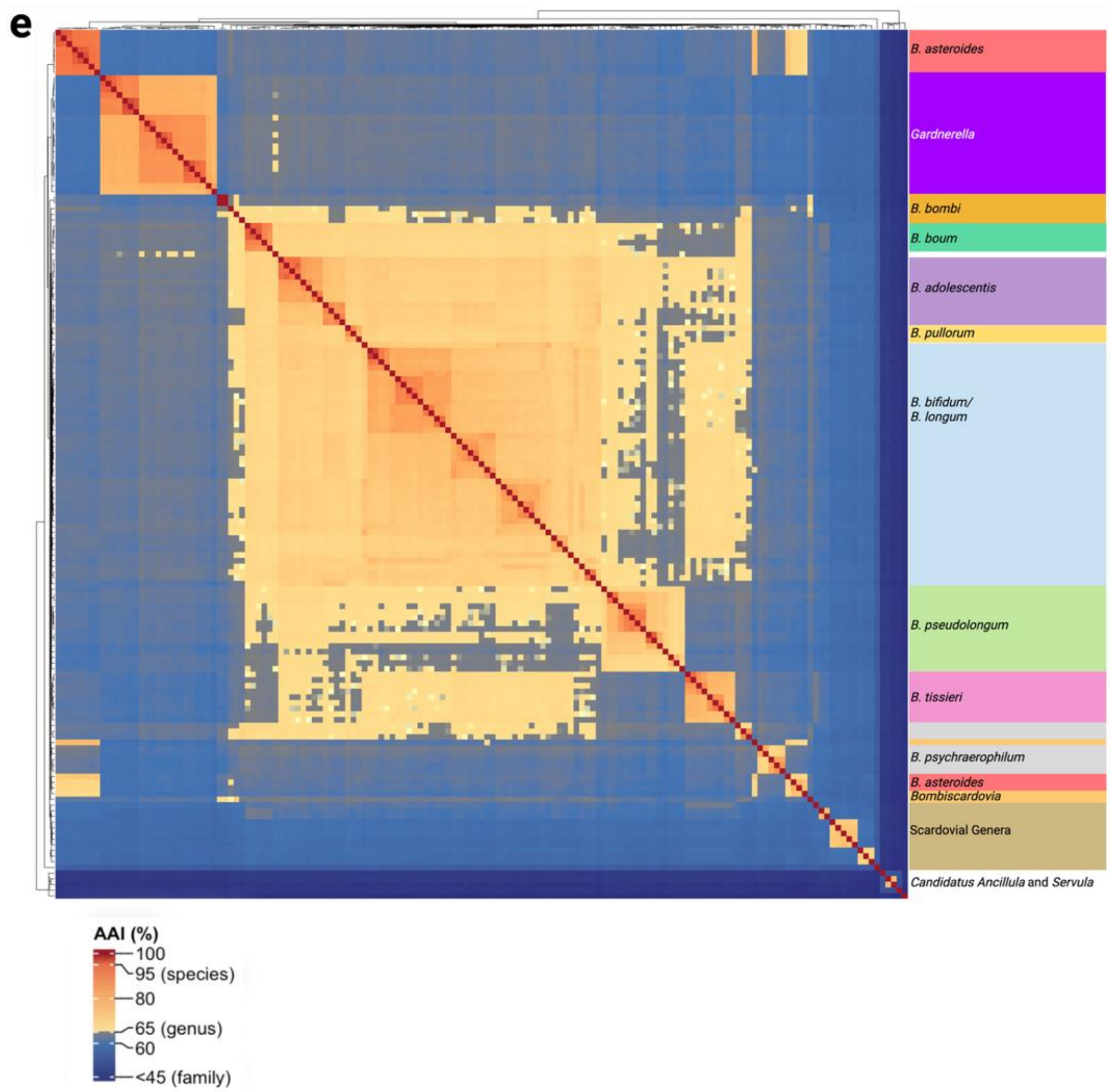
e
B. asteroides
Gardnerella
B. bombi
B. boum
B. adolescentis
B. pullorum
B. bifidum/
B. longum
B. pseudolongum
B. tissieri
B. psychraerophilum
B. asteroides
Bombiscardovia
Scardovial Genera
Candidatus Ancillula and Servula
AAI (%)
100
95 (species)
80
65 (genus)
60
<45 (family)

f

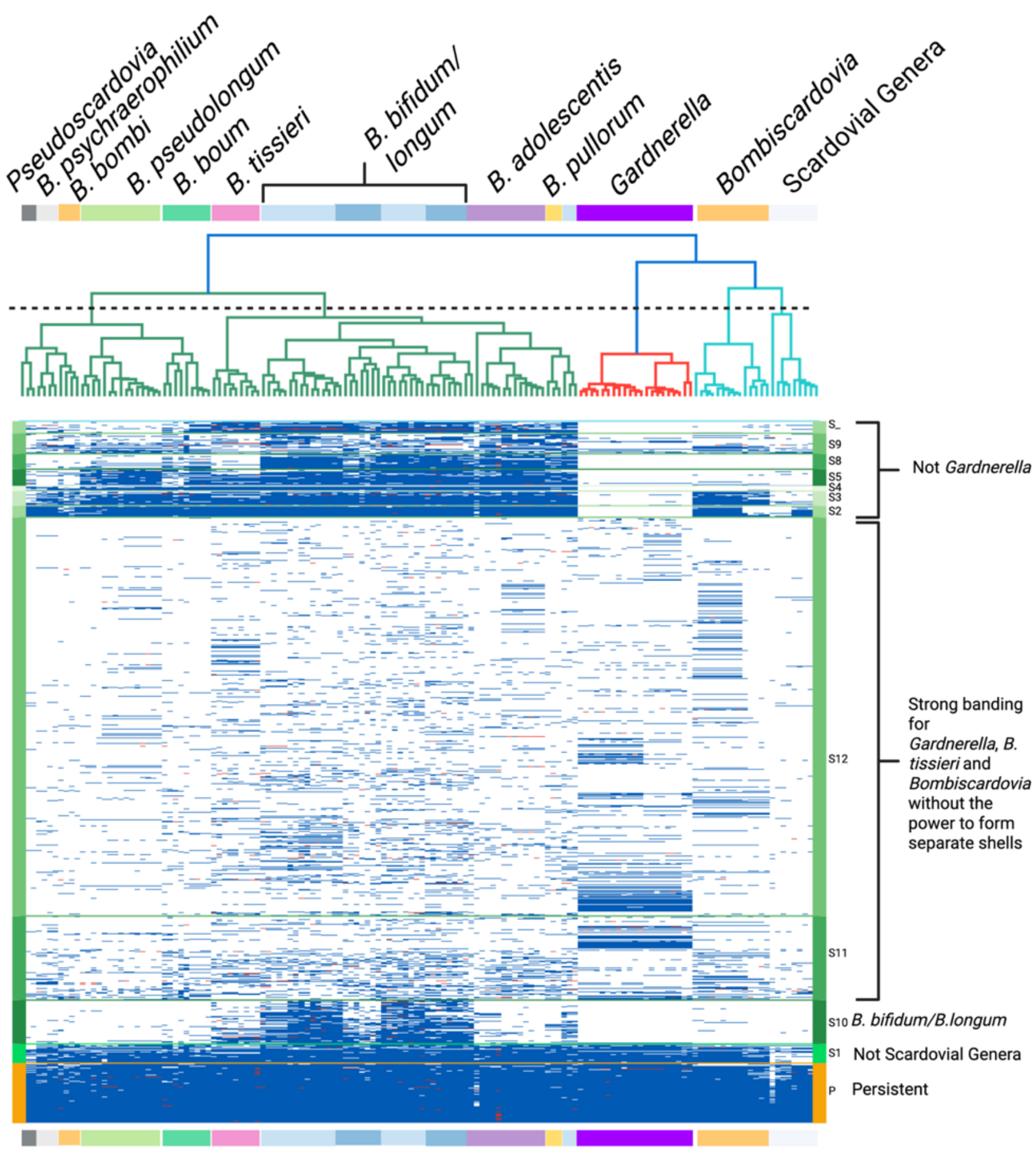

Supplementary Figure 1: Genomic comparisons among members of the family *Bifidobacteriaceae*

Comparative genomic analyses across representative genera of the family *Bifidobacteriaceae*, including subgroups of *Bifidobacterium* (as defined by Alessandri *et al.*), the "Scardovial" genera (e.g., *Aeriscardovia*, *Alloscardovia*, *Bombiscardovia*, *Galloscardovia*), and genera associated with termite gut microbiota (e.g., *Ancillula*, *Servulla*). **a,** Genome length comparisons across representative genera. Relative to *Bifidobacterium*, genomes of *Gardnerella* ($p < 0.001$, Kruskal–Wallis *post hoc* test with Bonferroni correction) and *Alloscardovia* ($p = 0.052$, Kruskal–Wallis *post hoc* with Bonferroni correction), are smaller. *Gardnerella* genomes are also significantly smaller than those of *Alloscardovia* ($p = 0.009$). Other comparisons involving Scardovial genera lack sufficient representation or exhibit broad variance that limits statistical inference. **b,** Genomic G+C content across selected genera. *Gardnerella*, *Ancillula*, and *Alloscardovia* exhibit significantly lower G+C percentages than *Bifidobacterium* ($p < 0.001$, $p = 0.0113$, and $p = 0.0179$, respectively. Kruskal–Wallis *post hoc* with Bonferroni correction). Bar plots were created in R. **c,** Digital DNA–DNA hybridization (dDDH) values among representative genomes. The species-level threshold (>70%) is highlighted in yellow; intermediate similarity (~40%) is shown in blue, and low similarity (<20%) in white. *Gardnerella* type strains form a monophyletic cluster within *Bifidobacteriaceae*. Groups marked with an asterisk indicate polyphyletic lineages. Colors along the axes correspond to the *Bifidobacterium* groups defined by Alessandri *et al.* Heatmap was created in R and annotated in BioRender. Bouzek, H. (2026) (https://BioRender.com/gsklyn8). **d,**

Conserved protein phylogeny of *Bifidobacteriaceae* type strains. Persistent PPanGGOLiN gene clusters were extracted and used to construct a neighbor-joining tree. *Gardnerella* forms a monophyletic lineage within the broader *Bifidobacteriaceae*, with the nearest cluster including a member of *Bombiscardovia*. Tree was created in iTOL. **e,** Amino acid identity (AAI) among type strains. Pairwise AAI values were calculated and visualized as a heatmap. Color gradients correspond to commonly applied genomic thresholds: dark blue (<45%, typical of inter-family divergence), light blue (45–65%, inter-generic range), yellow (>65%, genus-level coherence), and red (~100%, near-identical comparisons). Heatmap was created in R and annotated in BioRender. Bouzek, H. (2026) (https://BioRender.com/gsklyn8). **f,** PPanGGOLiN tile map of the pangenome derived from *Bifidobacteriaceae* type strains. Nodes (rows) correspond to gene clusters within the persistent fraction of the pangenome (orange bar). Shell partitions are indicated by green shades. The accompanying dendrogram is derived from a similarity matrix of gene presence–absence patterns. *Gardnerella* clusters with the Scardovial genera. The tile plot shows the relatively few persistent genes are uniquely shared between *Bifidobacterium* and *Gardnerella*. Tile plot created in PPanGGoLiN and annotated in BioRender. Bouzek, H. (2026) (https://BioRender.com/gsklyn8).

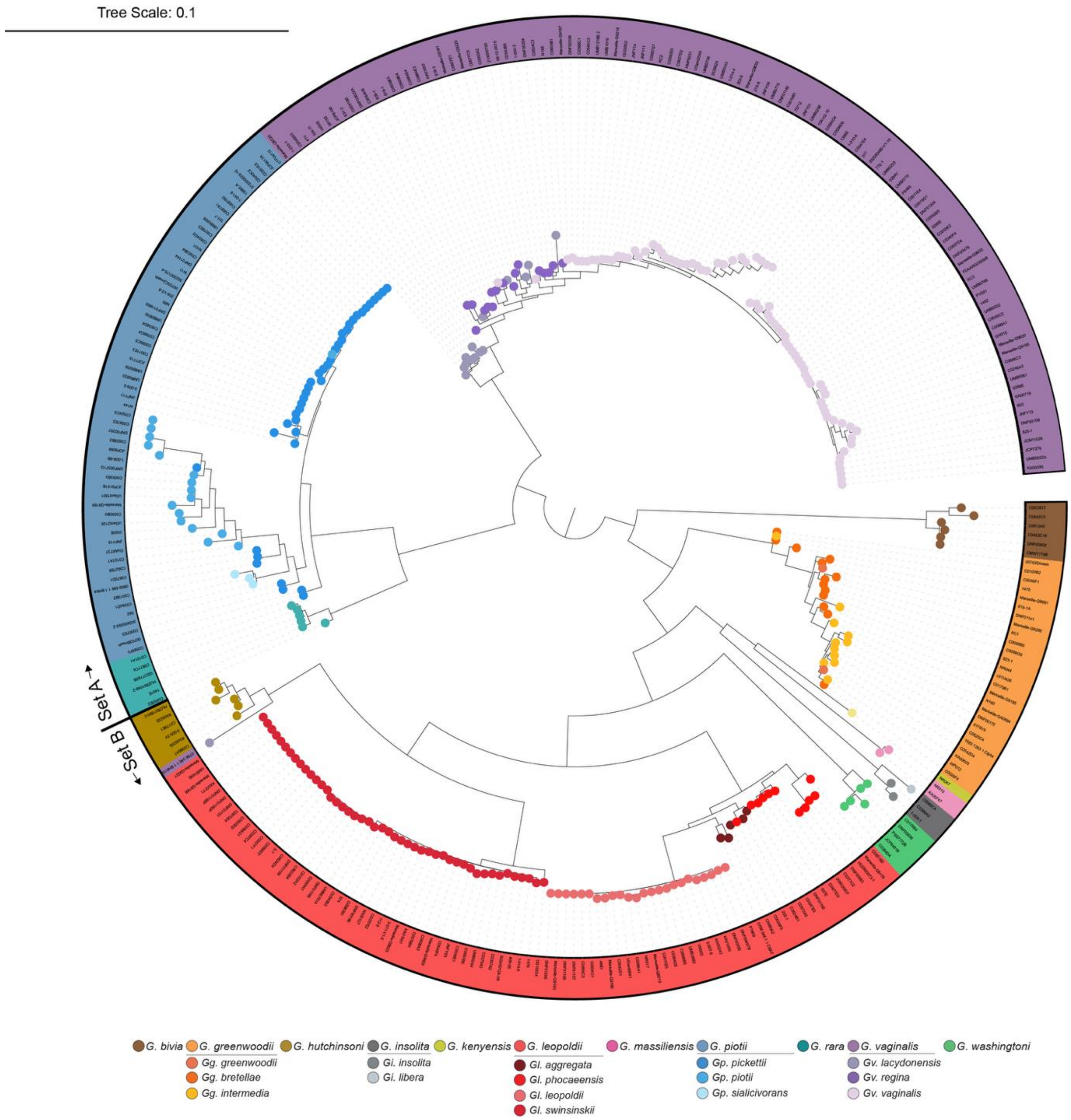

Supplementary Figure 2: *Gardnerella cpn60* gene sequence dendrogram

Maximum-likelihood dendrogram based on MUSCLE-aligned *cpn60* gene sequences from *Gardnerella* genomes. Label indicates strain identifier, while label and tip point color represent lineage. Sets are indicated by a division line and arrows pointing to included species. Dendrogram was created in iTOL and arranged in Adobe Illustrator.

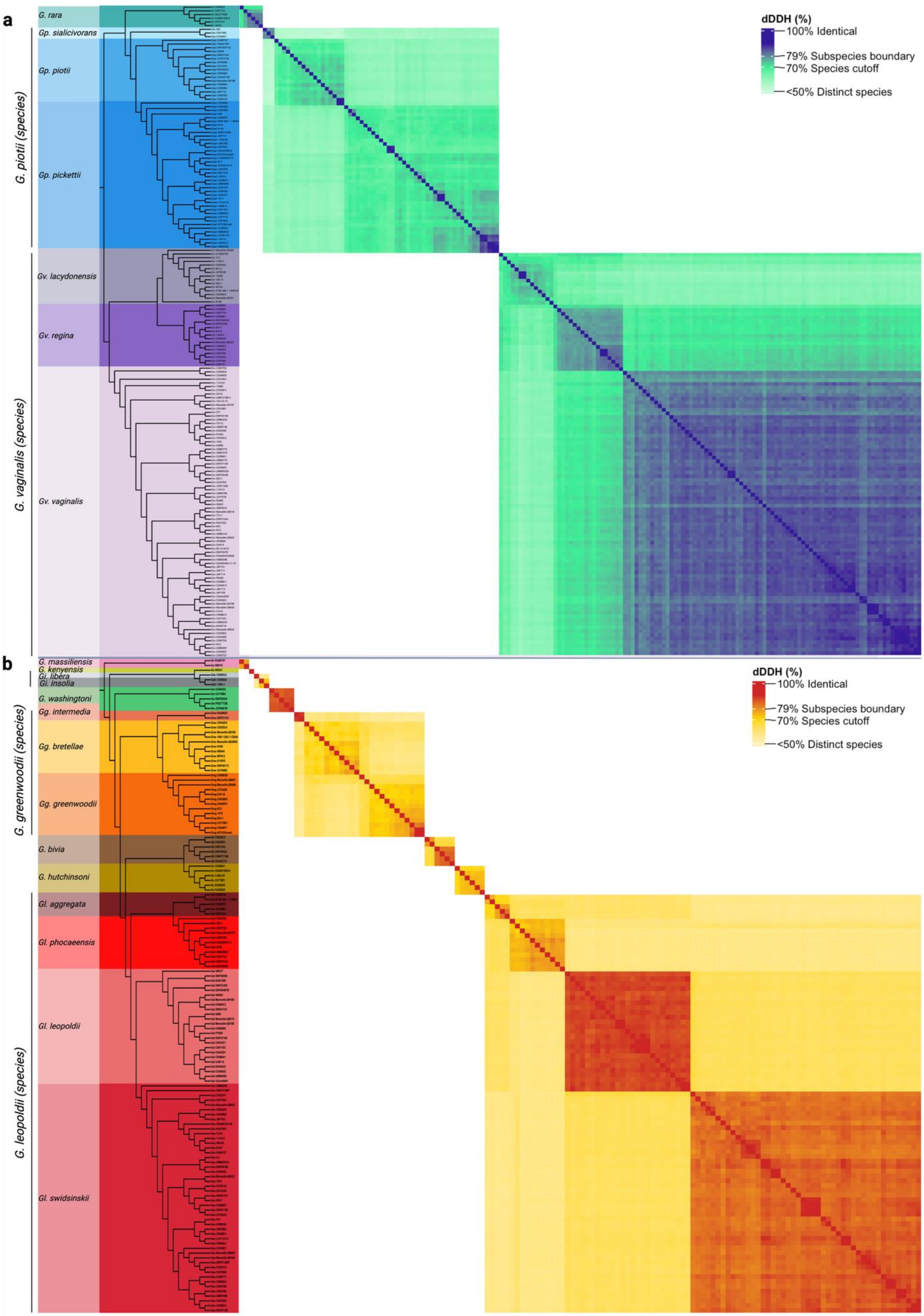
a
dDDH (%)
100% Identical
79% Subspecies boundary
70% Species cutoff
<50% Distinct species
G. rara
Gp. sialicivorans
Gp. piotii
Gp. pickettii
G. piotii (species)
Gv. lacydonensis
Gv. regina
Gv. vaginalis
G. vaginalis (species)
b
dDDH (%)
100% Identical
79% Subspecies boundary
70% Species cutoff
<50% Distinct species
G. massiliensis
G. kenyensis
Gl. libera
Gl. insolia
G. washingtoni
Gg. intermedia
Gg. bretellae
Gg. greenwoodii
G. greenwoodii (species)
G. bivia
G. hutchinsoni
Gl. aggregata
Gl. phocaeensis
Gl. leopoldii
Gl. swidsinskii
G. leopoldii (species)

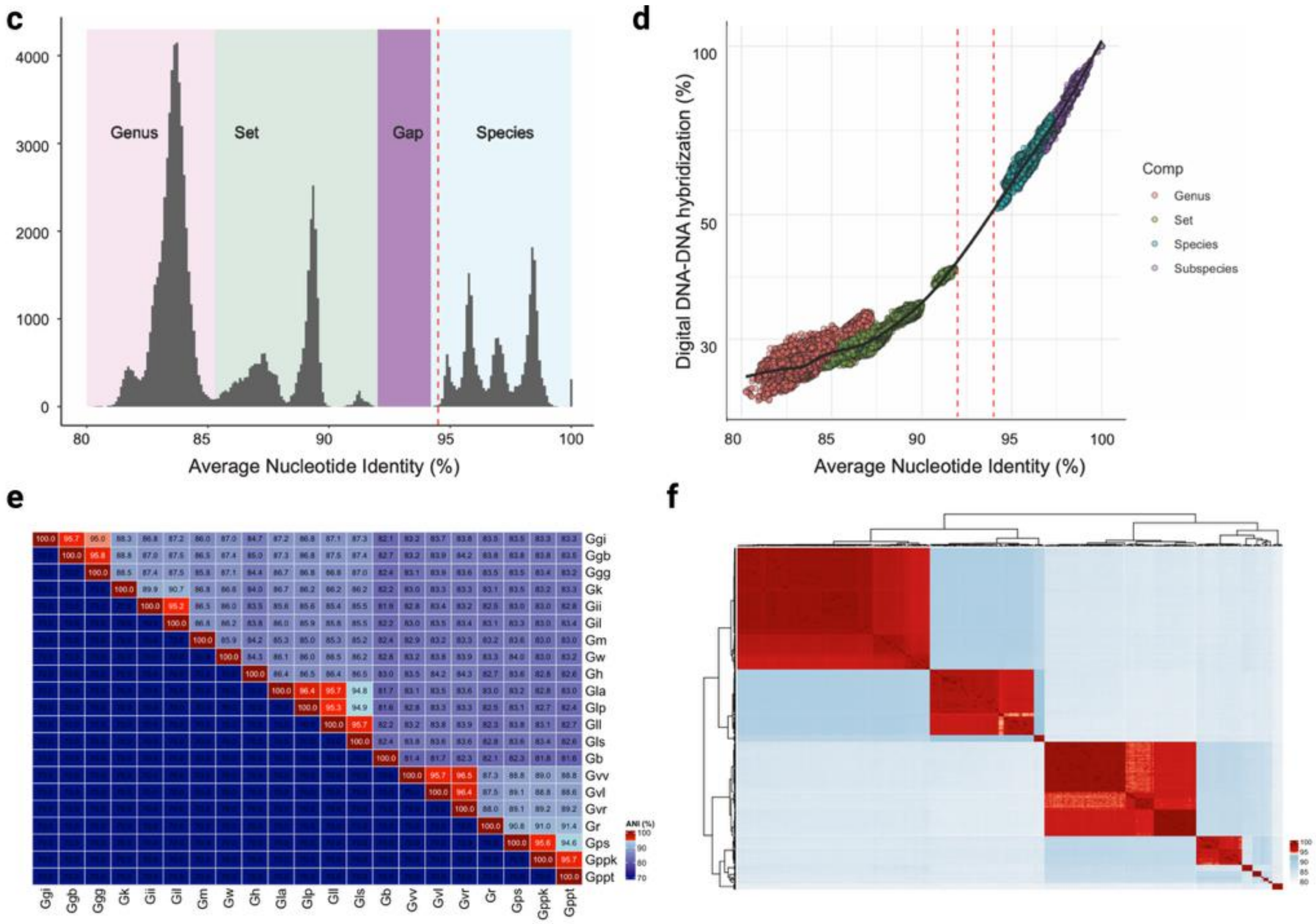
c
4000
3000
2000
1000
0
Genus
Set
Gap
Species
80
85
90
95
100
Average Nucleotide Identity (%)
d
Digital DNA-DNA hybridization (%)
100
50
30
Comp
Genus
Set
Species
Subspecies
80
85
90
95
100
Average Nucleotide Identity (%)
e
Ggi
Ggb
Ggg
Gk
Gii
Gil
Gm
Gw
Gh
Gla
Glp
Gll
Gls
Gb
Gvv
Gvl
Gvr
Gr
Gps
Gppk
Gppt
ANI (%)
100
90
80
70
f
100
95
90
85
80

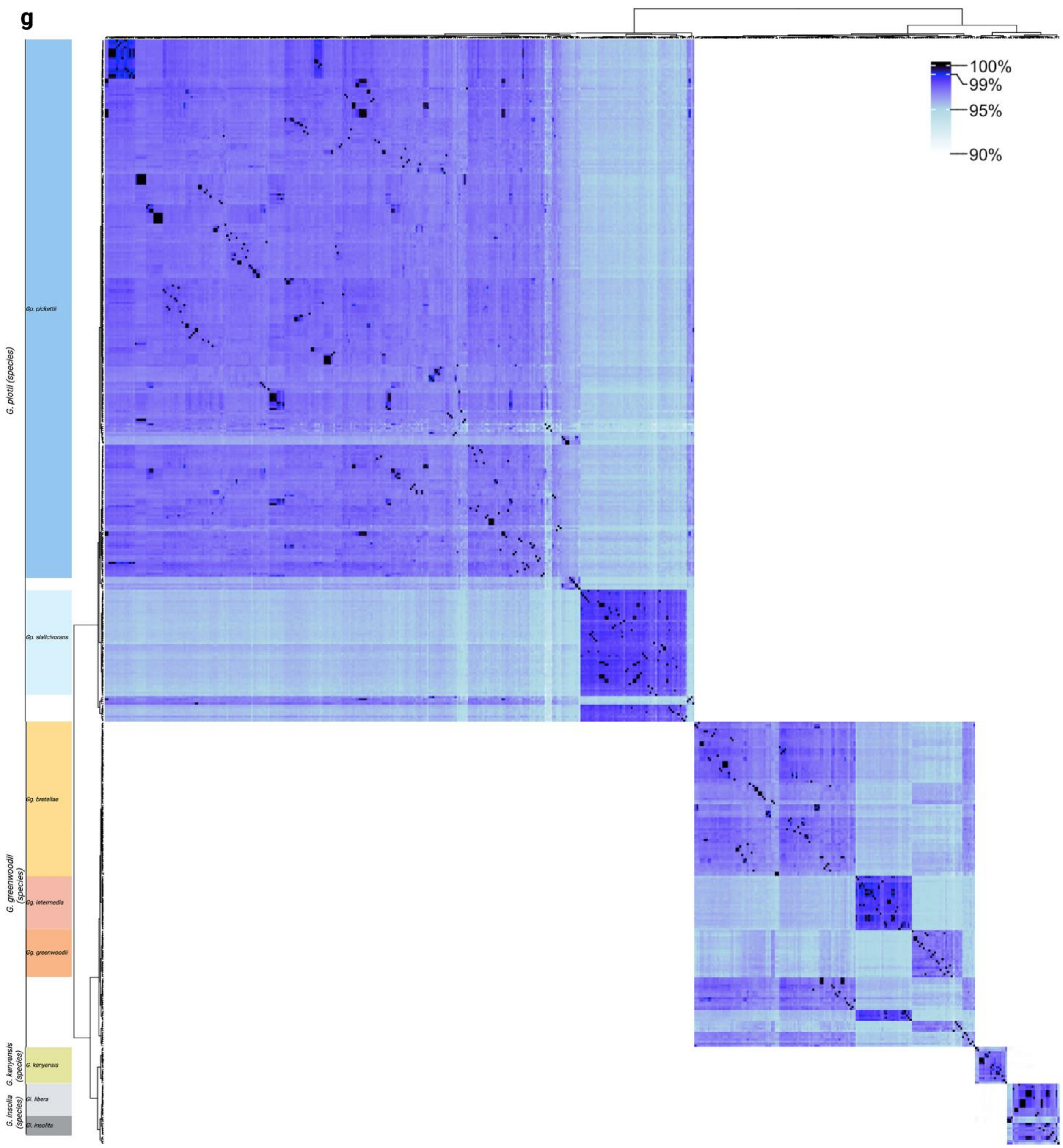

Supplementary Figure 3: Average nucleotide identity (ANI) and digital DNA-DNA hybridization (dDDH) comparisons between *Gardnerella* genomes.

a, dDDH comparison of the complete collection of Set A genomes as ordered by kSNP phylogeny **b**, dDDH comparison of the complete collection of Set B genomes as ordered by kSNP phylogeny. **c,** A histogram of the counts of ANI percentage comparisons between *Gardnerella* genomes showing the genus, set (subgenus), and species comparisons at each level. The gap between species and higher order comparisons falls slightly lower than 95% (red dashed line). **d**, Collinearity of ANI and dDDH values with red dashed lines showing the boundaries of the gap in both values. **e,** ANI of the type strains of *Gardnerella* species and subspecies. The mean of the pairwise ANI (i,j)

and (j,i) represented as just the upper triangle. Values from 100% to 95% are colored in increasingly lighter shades of red, values below 95.0% are colored in increasingly darker shades of blue. **f,** ANI of the *Gardnerella* strains in the curated collection with an emphasis on the division at 95% which is colored white. This emphasizes that the few strains that do fall in the 95-94% range are well within otherwise sharply defined species groups. **g**, Heatmap of pairwise ANI values for species in *Gardnerella* with low numbers of cultured isolates at the subspecies level as compared to MAGs (metagenome assembled genomes). Colored bands at the left give the species and subspecies groups for the boxes in the heatmap. Rows without coloration are to MAGs that are unassigned to species. Plots were created in R and annotated and arranged in BioRender. Bouzek, H. (2026) (https://BioRender.com/gsklyn8).

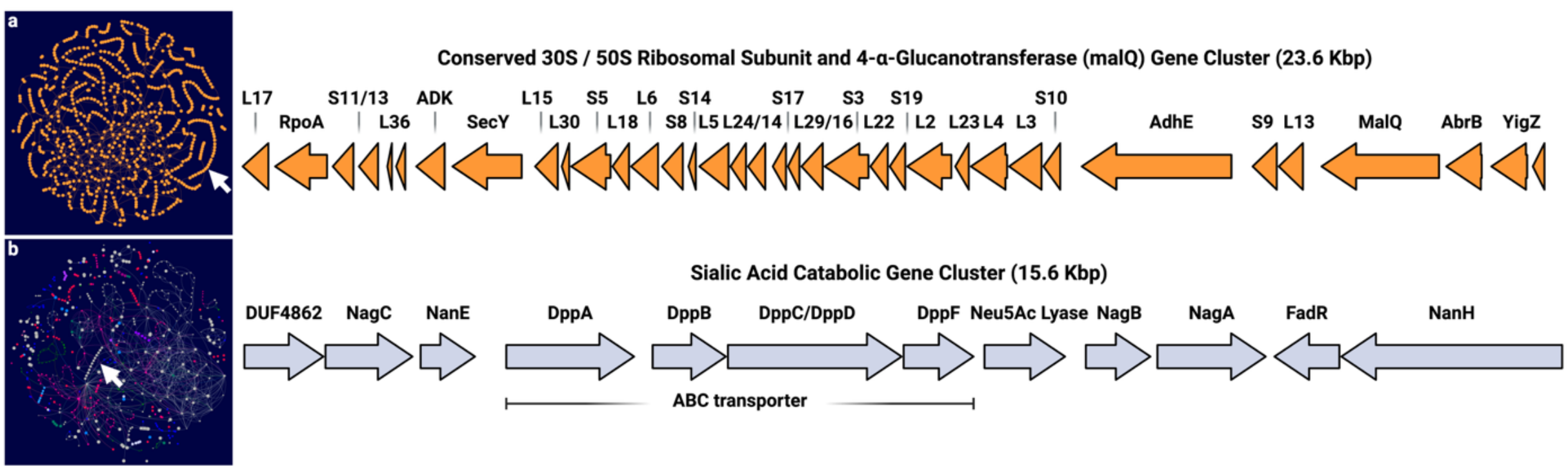

Supplementary Figure 4: PPanGGOLiN syntenic nodes create modules

a, Core and **b,** shell partitions from PPanGGOLiN-generated *Gardnerella* pangenome. Each node represents a gene group where the size of the node indicates relative presence across *Gardnerella* genomes. White arrows pointing to syntenic nodes represent neighboring genes in operons, shown on the right. Partition maps were created by PPanGGoLiN and Gephi. Heatmap was created using iTOL. Final figure was annotated and arranged in BioRender. Operon maps were created in BioRender. Bouzek, H. (2026) (https://BioRender.com/gsklyn8).

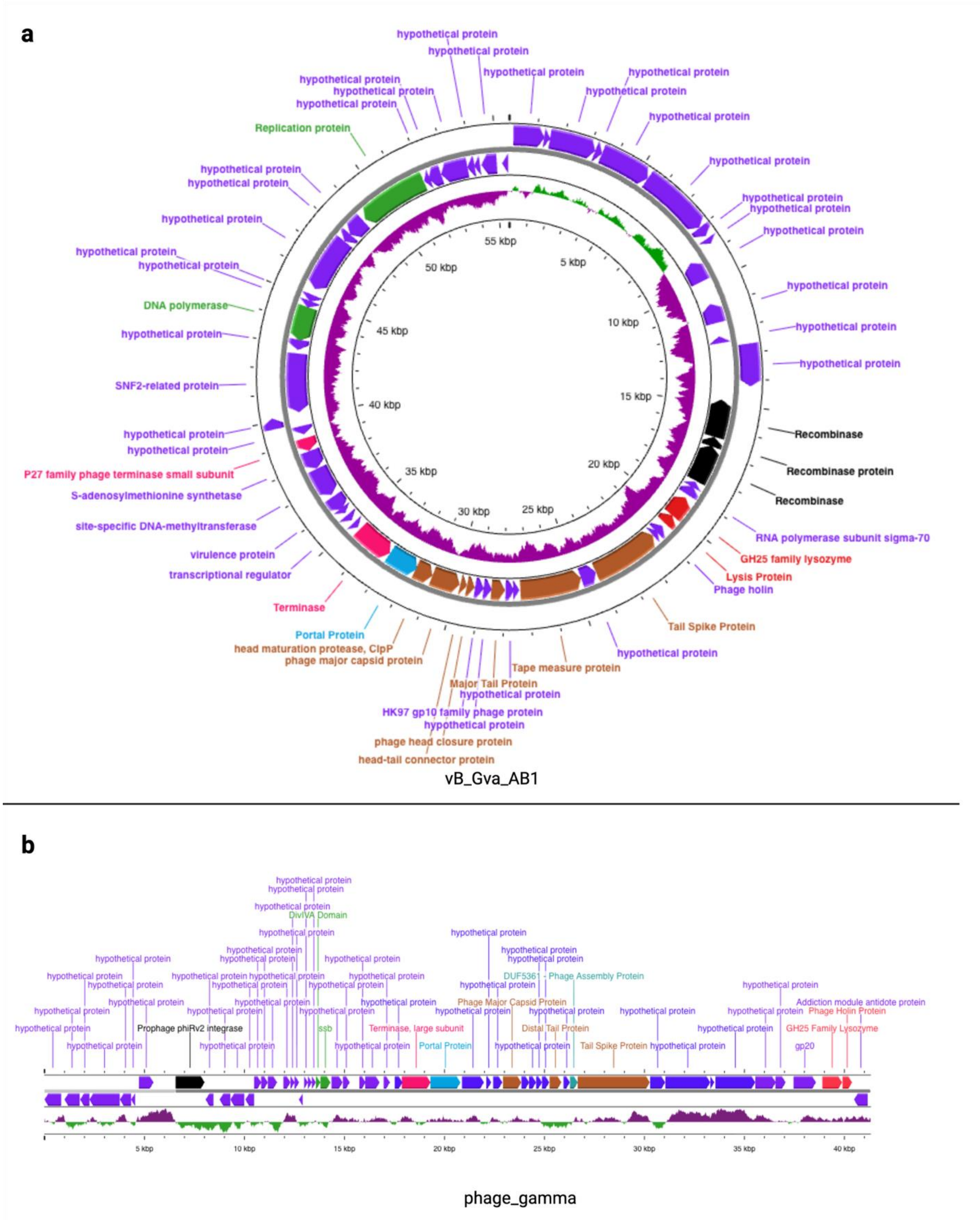
a
hypothetical protein
Replication protein
DNA polymerase
SNF2-related protein
P27 family phage terminase small subunit
S-adenosylmethionine synthetase
site-specific DNA-methyltransferase
virulence protein
transcriptional regulator
Terminase
Portal Protein
head maturation protease, ClpP
phage major capsid protein
Major Tail Protein
HK97 gp10 family phage protein
phage head closure protein
head-tail connector protein
Tape measure protein
Tail Spike Protein
Phage holin
Lysis Protein
GH25 family lysozyme
RNA polymerase subunit sigma-70
Recombinase
Recombinase protein
55 kbp
5 kbp
10 kbp
15 kbp
20 kbp
25 kbp
30 kbp
35 kbp
40 kbp
45 kbp
50 kbp
vB_Gva_AB1
b
hypothetical protein
DivIVA Domain
Prophage phiRv2 integrase
ssb
Terminase, large subunit
Portal Protein
DUF5361 - Phage Assembly Protein
Phage Major Capsid Protein
Distal Tail Protein
Tail Spike Protein
Addiction module antidote protein
Phage Holin Protein
GH25 Family Lysozyme
gp20
5 kbp
10 kbp
15 kbp
20 kbp
25 kbp
30 kbp
35 kbp
40 kbp
phage_gamma

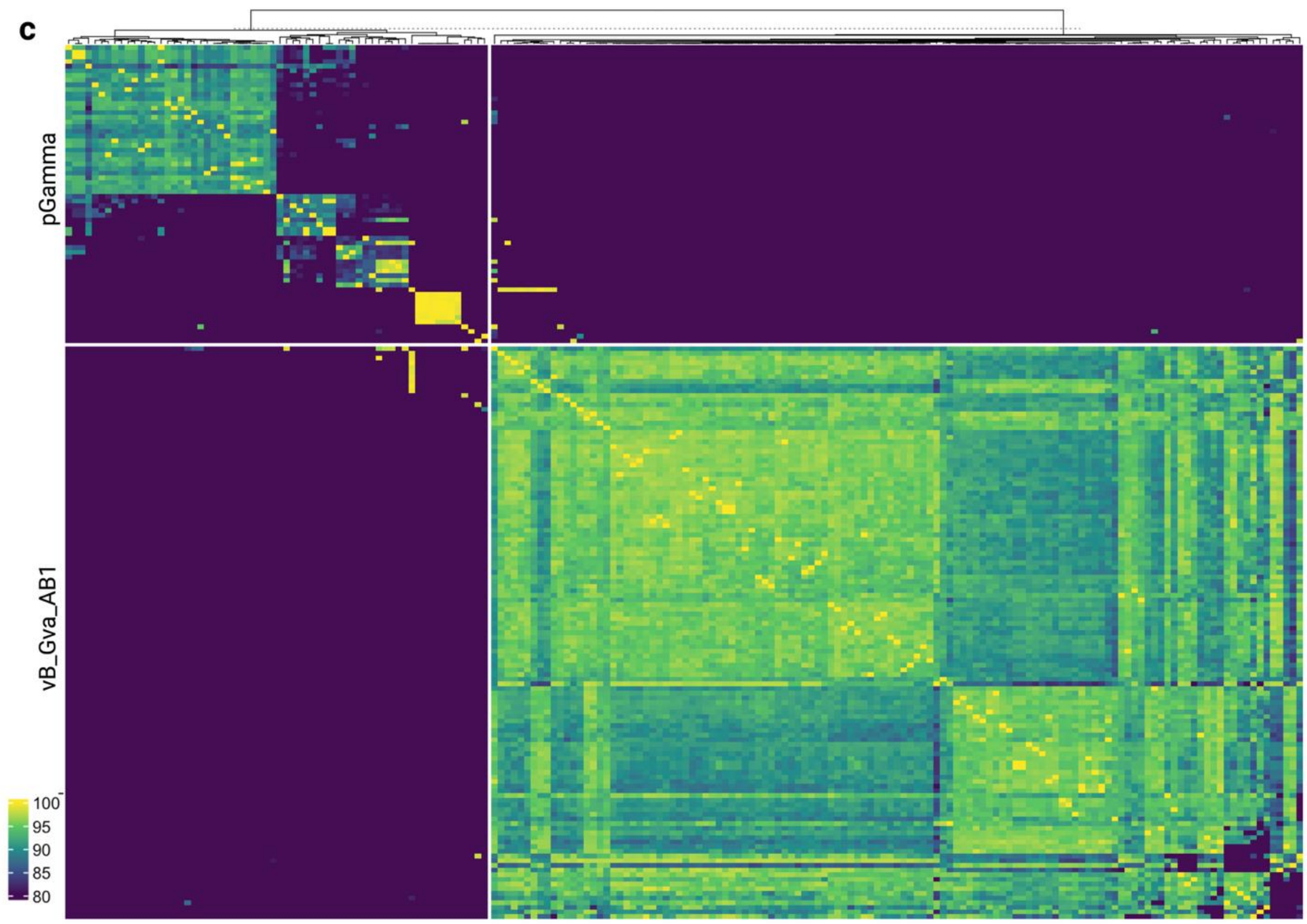

Supplementary Figure 5: Bacteriophages in *Gardnerella*

Phage maps of the different families of bacteriophage found in *Gardnerella*. **a,** Example of the vB_Gva_AB1 phage found in circular lytic form in *Gardnerella* strain 06-12-0010 and the lysogenic form of pGamma found in DNF00622A in **b,** Recombination genes are black, structural genes are brown, lysis/terminase genes are bright red, portal genes are blue, assembly genes are in green. and hypothetical genes are purple. The inner ring/bottom of the graph shows the GC% with high GC as purple and above the line while green means low GC and is below the line. **c,** ANI of the complete phages found in this collection. Clustering was by km=2 for the two phage types. The largest block closely aligns to vB_Gva_AB1 phage. The smaller group made up of >3 smaller subgroups. The group that forms the strongest cohesion (yellow box in pGamma) are all from a single lab in China (Zhang 2022) and may overrepresent the natural occurrence of phage of this type. Plots were created in Proksee and R and arranged in in BioRender. Bouzek, H. (2026) https://BioRender.com/gsklyn8.

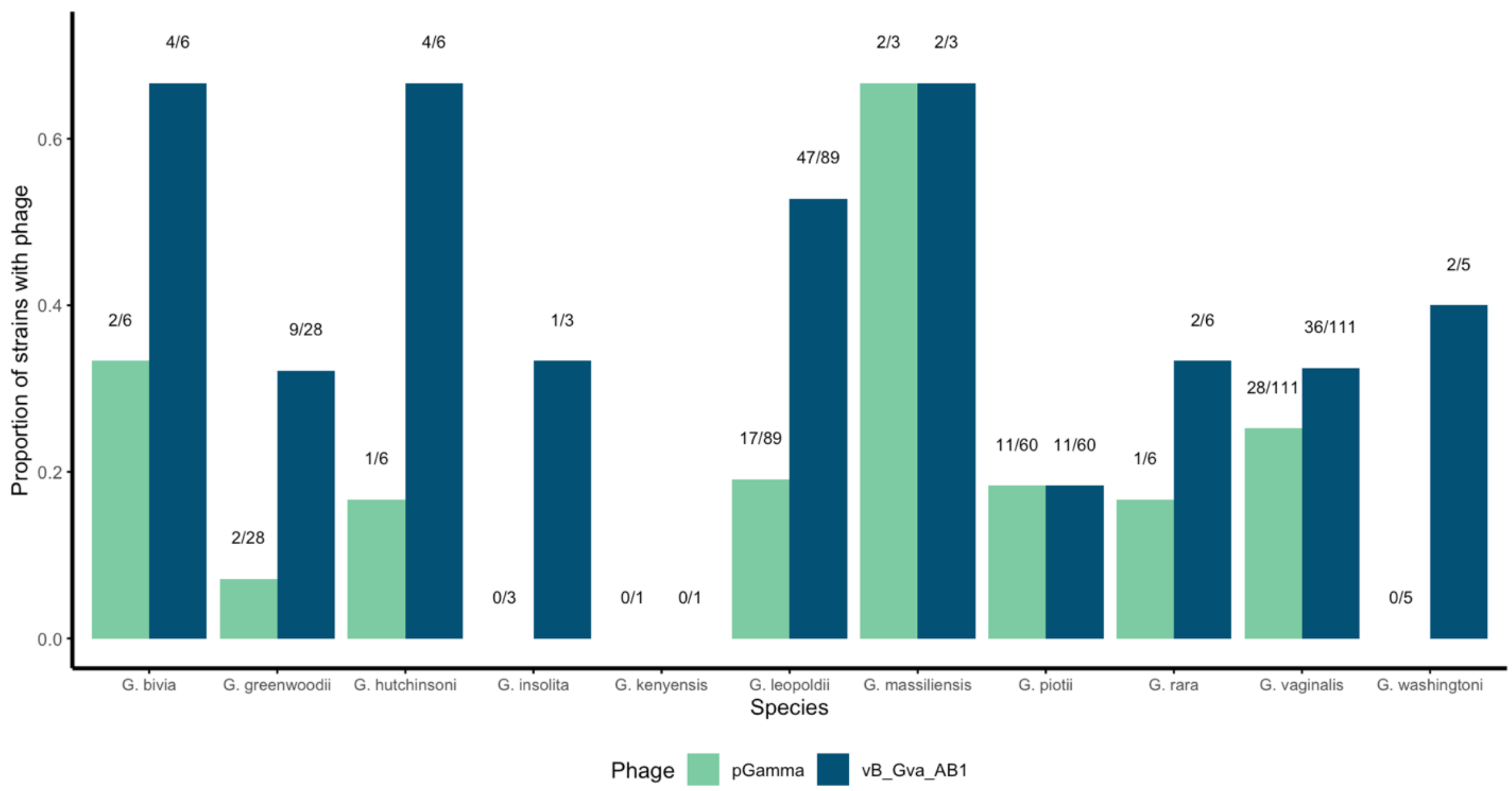

Supplementary Figure 6: Phage Genomes

Graphs show the proportion number of intact prophages detected in each species. Prophages were found alone or in pairs. Overall, infection probability differed significantly by phage type. Relative to pGamma, phage vB_Gva_AB1 was associated with 2.34-fold higher odds of infection (OR = 2.34, 95% CI 1.63–3.43, $p < 0.001$; logistic regression). No significant overall effect of host species on infection probability was detected after accounting for phage type. Species coefficients were not individually significant in the additive logistic model after correcting for phage effects, although G. piotii approached significance ($p = 0.058$) with a lower infection rate.

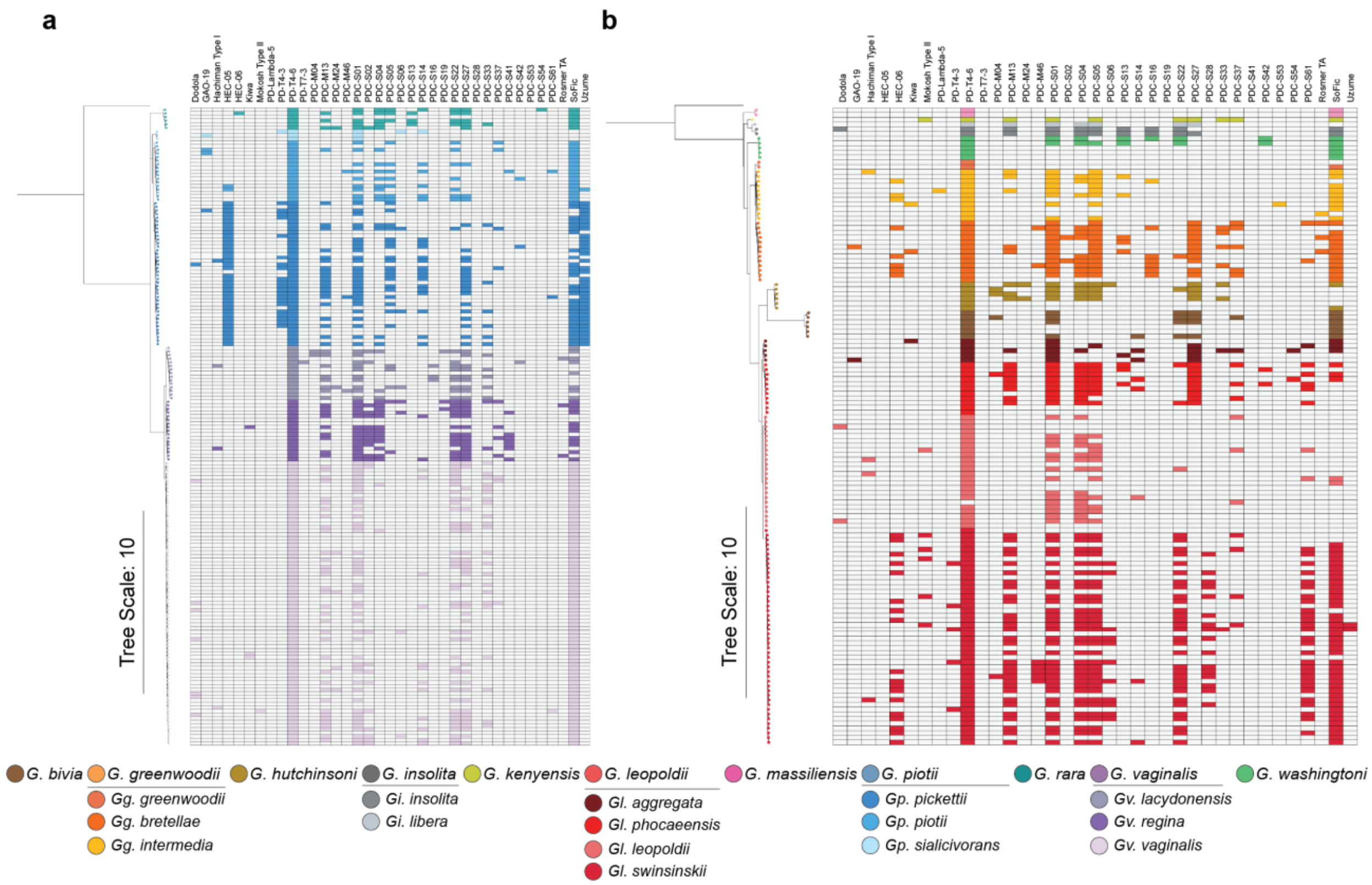

Supplementary Figure 7: Predicted genomic defenses with undescribed mechanisms PADLOC-predicted defenses with currently undescribed mechanisms of action across *Gardnerella* genomes in **a,** Set A or **b,** Set B. Genomes are ordered by kSNP4 phylogenies, with colors indicating lineage. Heatmaps were created in iTOL and arranged in Adobe Illustrator.

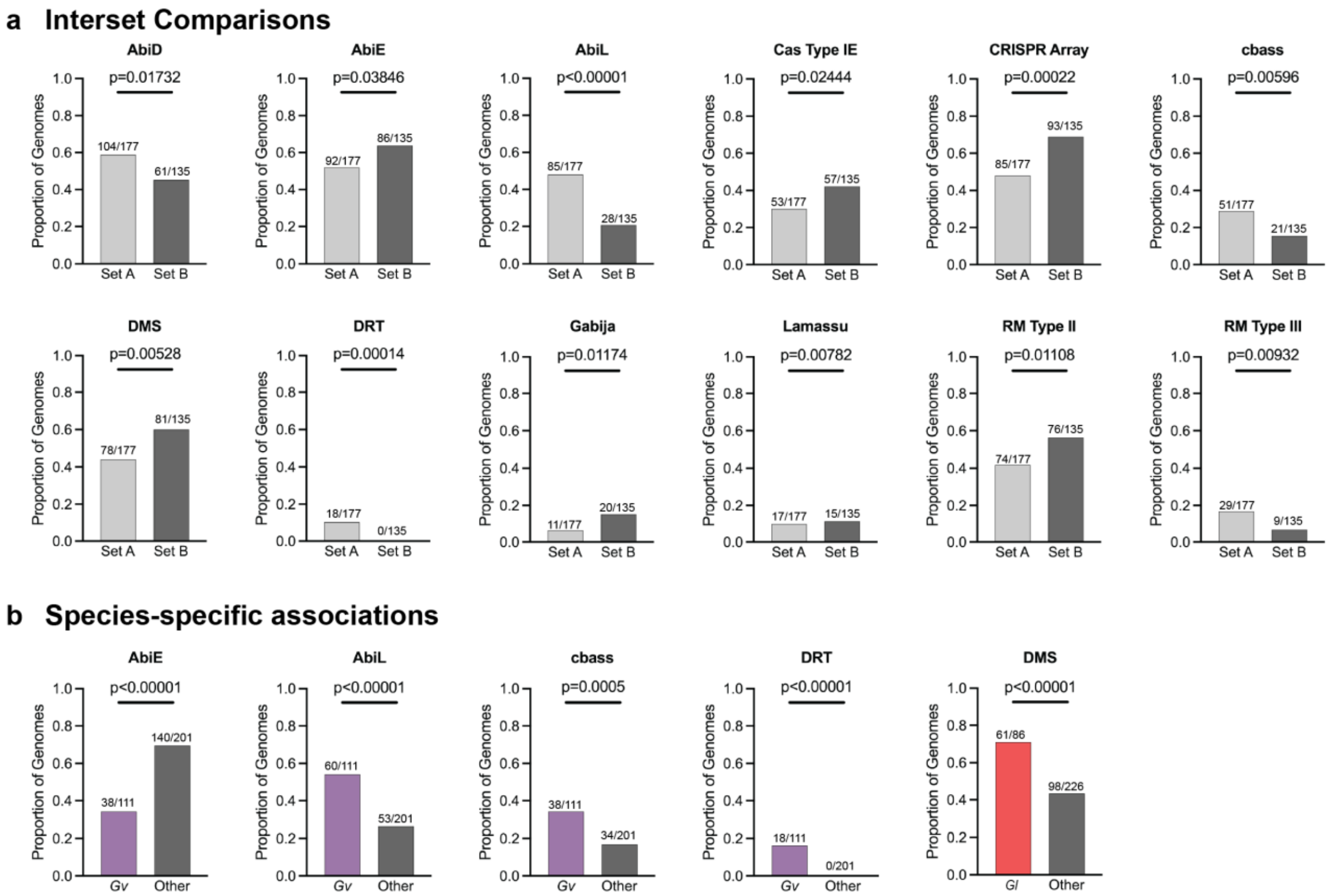

Supplementary Figure 8: Genetic defense system associations across *Gardnerella* groups Graphs show **a,** the presence of PADLOC-predicted defenses compared by genome proportion between *Gardnerella* Set A and Set B and **b,** specific species, and all other species. Statistical analysis was carried out using a two-sample z-test, two-tailed. The bar plots were generated in GraphPad Prism (v10.3.1) and arranged in Adobe Illustrator.

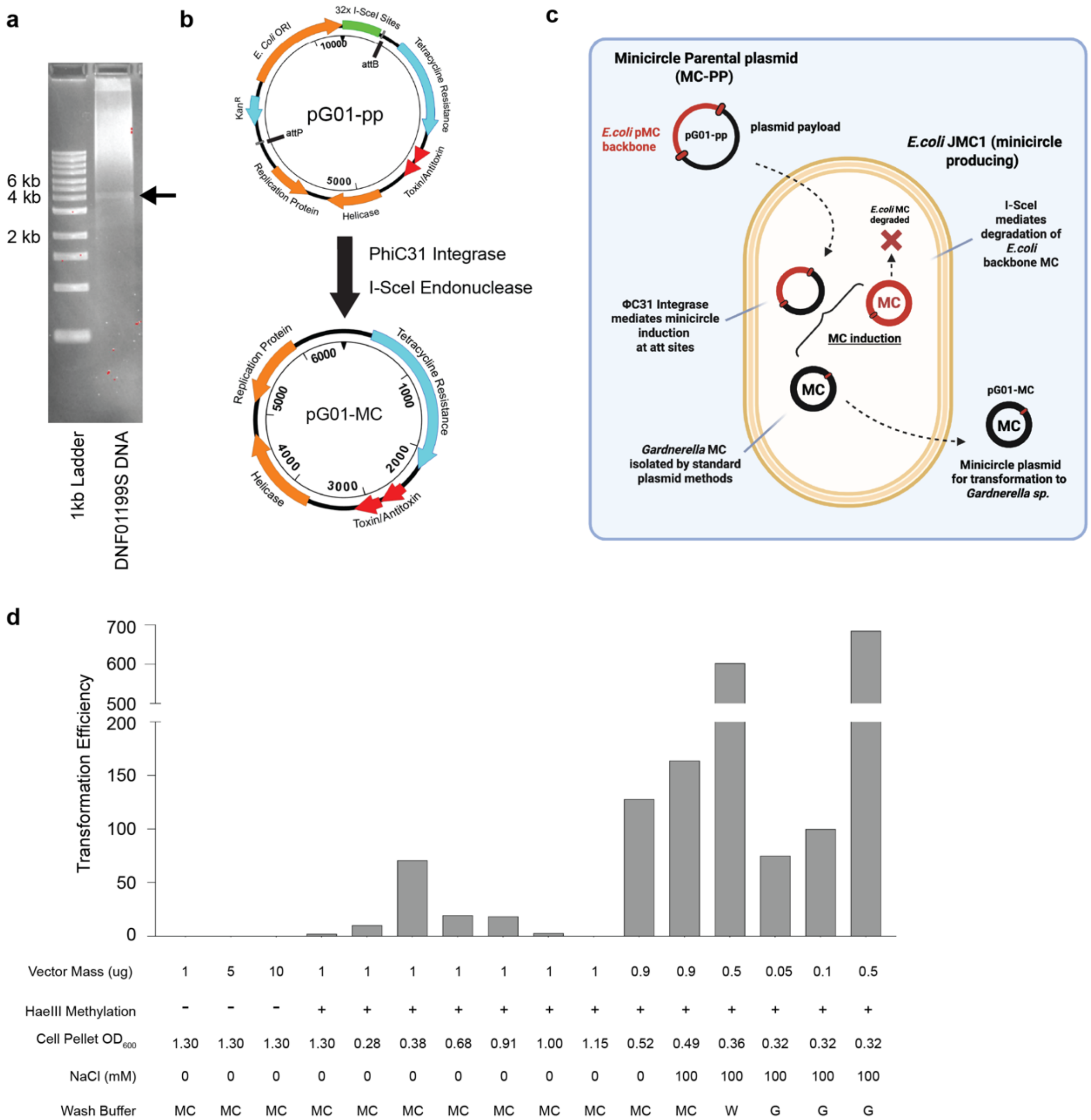

Supplementary Figure 9: Transformation in *Gardnerella* strain ATCC 14018 of a vector tool derived from a native *Gardnerella* plasmid

a, pDNF0199S-01, a 4,028 bp plasmid, isolated from *Gardnerella* strain DNF01199S. **b-c,** Schematic shows the components of pG01-pp, **b,** the minicircle parental plasmid derived from pDNF01199S-01, and **c,** the resulting pG01-MC produced after minicircle induction. **d,** Transformation efficiency of pG01-MC in *Gardnerella* strain ATCC 14018 under different conditions, including changes to vector mass, the pre-methylation of pG01-MC with *HaeIII* to methyl-modify GGCC sites, the OD_{600} of the cell pellet used, the concentration of sodium chloride (NaCl) present in the cell growth media, and the wash buffer used during electrocompetent cell preparation (MC=maltose citrate, W=milliQ water, G=10% glycerol). The gel is from a image that was cropped to include only the pertinent lanes, the plasmid drawing was created in BioRender. Zepeda, M. (2026) https://BioRender.com/ocsjlp2, and the bar chart was made in R. The figures were arranged in Adobe Illustrator.
