# Supplementary material for: A syntenic pangenome of Gardnerella reveals novel plasmids and phage, taxonomic boundaries, and species-level stratification of metabolic and virulence potential
Source: Nat Commun. 2026 Jun 20;17:8510. doi: 10.1038/s41467-026-74458-2 (PMC13483204; doi:10.1038/s41467-026-74458-2)
Supplement: Supplementary file 3 [file 41467_2026_74458_MOESM4_ESM.pdf]

nature portfolio

Corresponding author(s): CHRISTOPHER JOHNSTON

Last updated by author(s): 2026/05/11

Reporting Summary

Nature Portfolio wishes to improve the reproducibility of the work that we publish. This form provides structure for consistency and transparency in reporting. For further information on Nature Portfolio policies, see our Editorial Policies and the Editorial Policy Checklist.

Statistics

For all statistical analyses, confirm that the following items are present in the figure legend, table legend, main text, or Methods section.

n/a	Confirmed	
☐	☒	The exact sample size (*n*) for each experimental group/condition, given as a discrete number and unit of measurement
☒	☐	A statement on whether measurements were taken from distinct samples or whether the same sample was measured repeatedly
☐	☒	The statistical test(s) used AND whether they are one- or two-sided<br>*Only common tests should be described solely by name; describe more complex techniques in the Methods section.*
☒	☐	A description of all covariates tested
☒	☐	A description of any assumptions or corrections, such as tests of normality and adjustment for multiple comparisons
☐	☒	A full description of the statistical parameters including central tendency (e.g. means) or other basic estimates (e.g. regression coefficient) AND variation (e.g. standard deviation) or associated estimates of uncertainty (e.g. confidence intervals)
☐	☒	For null hypothesis testing, the test statistic (e.g. *F*, *t*, *r*) with confidence intervals, effect sizes, degrees of freedom and *P* value noted<br>*Give P values as exact values whenever suitable.*
☒	☐	For Bayesian analysis, information on the choice of priors and Markov chain Monte Carlo settings
☒	☐	For hierarchical and complex designs, identification of the appropriate level for tests and full reporting of outcomes
☒	☐	Estimates of effect sizes (e.g. Cohen's *d*, Pearson's *r*), indicating how they were calculated

Our web collection on statistics for biologists contains articles on many of the points above.

Software and code

Policy information about availability of computer code

Data collection	n/a
Data analysis	n/a

For manuscripts utilizing custom algorithms or software that are central to the research but not yet described in published literature, software must be made available to editors and reviewers. We strongly encourage code deposition in a community repository (e.g. GitHub). See the Nature Portfolio guidelines for submitting code & software for further information.

Data

Policy information about availability of data

All manuscripts must include a data availability statement. This statement should provide the following information, where applicable:

- Accession codes, unique identifiers, or web links for publicly available datasets
- A description of any restrictions on data availability
- For clinical datasets or third party data, please ensure that the statement adheres to our policy

The genome data generated in this study have been deposited in the NCBI database under BioProject numbers PRJNA639145, PRJNA797778, PRJNA798061, PRJNA762089, PRJNA1224438, and PRJNA1347778. Additional genome data utilized in this study are provided in the Supplementary Table 1 and Source Data file.

Research involving human participants, their data, or biological material

Policy information about studies with human participants or human data. See also policy information about sex, gender (identity/presentation), and sexual orientation and race, ethnicity and racism.

Reporting on sex and gender	N/A
Reporting on race, ethnicity, or other socially relevant groupings	N/A
Population characteristics	N/A
Recruitment	N/A
Ethics oversight	*Identify the organization(s) that approved the study protocol.*

Note that full information on the approval of the study protocol must also be provided in the manuscript.

Field-specific reporting

Please select the one below that is the best fit for your research. If you are not sure, read the appropriate sections before making your selection.

[x] Life sciences   [ ] Behavioural & social sciences   [ ] Ecological, evolutionary & environmental sciences

For a reference copy of the document with all sections, see nature.com/documents/nr-reporting-summary-flat.pdf

Life sciences study design

All studies must disclose on these points even when the disclosure is negative.

Sample size	N/A
Data exclusions	Genome completeness and contamination for all sequenced and publicly available Gardnerella genomes retrieved from NCBI were assessed using CheckM (v 1.0.18). All genomes were annotated via RAST (v 1.073). Genomes with contamination greater than 1.5% or completeness below 90% were excluded from downstream analyses.<br>Redundant genome sequences were identified based on pairwise average nucleotide identity (ANI). Genomes sharing > 99.5% ANI with other genomes in the collection were considered duplicates and a single representative genome was retained for each duplicate group (Supplementary Data 1). Pairwise average nucleotide identity (ANI) metrics were calculated separately for Set A and Set B genomes using FastANI (v1.34) and visualized using the R packages ComplexHeatmap (v 2.27.0). Digital DNA-DNA hybridization (dDDH) values were calculated via the Genome-to-Genome Distance Calculator (v 3.0) and visualized in R (v 4.5.1 -- 2025-06-13 -- "Great Square Root"). Amino Acid Identity (AAI) values for Bifidobacteriaceae type strains were determined using pairwise comparisons of FastAAI (version 2).
Replication	N/A
Randomization	N/A
Blinding	N/A

Reporting for specific materials, systems and methods

We require information from authors about some types of materials, experimental systems and methods used in many studies. Here, indicate whether each material, system or method listed is relevant to your study. If you are not sure if a list item applies to your research, read the appropriate section before selecting a response.

Materials & experimental systems

n/a	Involved in the study	
[x]	[ ]	Antibodies
[x]	[ ]	Eukaryotic cell lines
[x]	[ ]	Palaeontology and archaeology
[x]	[ ]	Animals and other organisms
[x]	[ ]	Clinical data
[x]	[ ]	Dual use research of concern
[x]	[ ]	Plants

Methods

n/a	Involved in the study	
[x]	[ ]	ChIP-seq
[x]	[ ]	Flow cytometry
[x]	[ ]	MRI-based neuroimaging

Plants

Seed stocks	N/A
Novel plant genotypes	N/A
Authentication	N/A
