# Supplementary material for: A syntenic pangenome of Gardnerella reveals novel plasmids and phage, taxonomic boundaries, and species-level stratification of metabolic and virulence potential
Source: Nat Commun. 2026 Jun 20;17:8510. doi: 10.1038/s41467-026-74458-2 (PMC13483204; doi:10.1038/s41467-026-74458-2)
Supplement: Supplementary file 4 [file 41467_2026_74458_MOESM5_ESM.pdf]

nature portfolio

Peer Review File

A Syntenic Pangenome of *Gardnerella* Reveals Novel Plasmids and Phage, Taxonomic Boundaries, and Species-Level Stratification of Metabolic and Virulence Potential

Corresponding Author: Dr Christopher Johnston

Parts of this Peer Review File have been redacted as indicated to remove third-party material.

This file contains all reviewer reports in order by version, followed by all author rebuttals in order by version.

Version 0:

Reviewer comments:

Reviewer #1

(Remarks to the Author)
Bouzek et al. describe the sequencing and bioinformatic analysis of 392 Gardnerella isolates. A new taxonomy for Gardnerella species is proposed based on these analyses. Impactful discoveries were made here, including the description of a new phage and the identification of cryptic plasmids in some strains. Furthermore, an E. coli-Gardnerella shuttle plasmid was created and used to transform Gardnerella vaginalis.
1. This study is a tour-de-force analysis of Gardnerella genomics and will be highly useful to the field.
2. The paragraph describing the evolution of the naming conventions for Gardnerella (lines 80-102) is overwrought. The recognition that Gardnerella are multiple different species has been clarifying for the field and has allowed it to move on from Ahmed's clade designations to actual species names and the ability to characterize those species. Thus, the authors should tone-down their description of the confusion brought about by the multiple attempts to accurately describe the diversity of Gardnerella. In particular, the claim that the designation of species is compounding the complexity (line 100) should be removed, especially since the authors in this study are designating new species in the Gardnerella genus.
3. The taxonomy rules for naming prokaryotic species are somewhat complex, but I do not think the current accepted names for Gardnerella species can simply be discarded as proposed in lines 121-125 and Fig 1 and Fig 2. I refer the authors to the International Code of Nomenclature of Prokaryotes. Prokaryotic Code (2022 Revision) (doi.org/10.1099/ijsem.0.005585). In particular, on the subject of priority, I think the names for three of the species will have to be retained (Chapter 3, Section 5, Rule 23a). The authors propose the species G. aggregata, but it contains the named species G. leopoldii and G. swidsinskii. According to the Code, the authors can choose one of those to be the new species name. Similarly, the proposed G. sialicivorans would need to be either G. piottii or G. pickettii. The proposed new species G. media would need to be G. greenwoodii. Also, I would encourage the authors not to choose "media" as a species name since searches for "Gardnerella media" are likely to return lots of hits for NYCIII and HBT plates and not the desired information about the bacteria.
4. The claim that "variable lineage-dependent pathogenic potential exists across Gardnerella" (lines 211-212) is an over-interpretation of the data presented. The statement implies that all the species and subspecies are or can be pathogens, and the bioinformatic analysis is not sufficient to make that claim. Perhaps the authors meant something else and just need to state that more specifically.
5. The identification of a new phage in Gardnerella (phage gamma) is exciting (line 257). The authors should include a figure showing the phage gamma gene map from a representative strain.

Minor points.
1. Line 72. Correct spelling to "Scardovia".
2. Contrary to what is stated in the text (line 105), Extended data Fig. 2 does not indicate which species are Set A and Set B.
3. The presence of tetM in Gardnerella providing tetracycline resistance was published and should be cited in line 330. Roberts MC et al. 1986 (doi: 10.1128/AAC.30.5.810).
4. Only report significant figures for the transformation efficiency (line 346).

Reviewer #2

(Remarks to the Author)
Heater Bouzek and colleagues present a comprehensive genome analysis of Gardnerella species, leveraging a high-quality reference set of genomes derived from both publicly available data and newly sequenced isolates. Through a robust bioinformatics approach, they identified novel genomic lineages that may represent new species and subspecies of Gardnerella. Notably, the team constructed the first syntenic Gardnerella pangenomes, uncovering lineage-specific virulence factors, metabolic pathways, and defense systems. They also identified native plasmids and demonstrated their potential utility as molecular tools. Collectively, this study establishes a complete genomic and functional framework for the classification and mechanistic investigation of Gardnerella species, providing a valuable foundation for future bacterial vaginosis research.

The study is well-designed and clearly analyzed and presented. The scientific foundation is valid, and my questions and suggestions pertain only to modest improvements in the analysis and presentation.

1. The classification of Gardnerella within the family Bifidobacteriaceae remains unresolved. Multiple phylogenetic analyses have consistently positioned members of this genus within Bifidobacterium (https://doi.org/10.1128/AEM.02188-20, https://doi.org/10.1111/eva.12555, https://doi.org/10.1186/s12864-017-3955-4), suggesting that reclassification may be warranted. This proposed reclassification is reflected in the GTDB (as acknowledged in your manuscript). However, no formal taxonomic proposals have yet been made, and the genus name Gardnerella continues to be valid and recognized in current nomenclature.
In lines 77–79, the authors state that Gardnerella and Bifidobacterium are distinct genera, basing this claim on differences in G+C content, genome length, and—surprisingly—an ANI threshold of 73–75%. Is the ANI threshold for genus-level demarcation universally accepted? To date, ANI thresholds have only been formally established for species-level delineation (95%).
Regarding the presented heatmap of ANI values (Extended Data Fig. 1c), it is unclear whether the clustering was derived from phylogenetic placements or hierarchical clustering of ANI values. To strengthen the analysis, I recommend including a genome-based phylogenetic tree of all available type strains within Bifidobacteriaceae. This would allow verification of the phylogenetic placement of Gardnerella species and clarify their distinction from Bifidobacterium.

2. The authors applied an ANI value of 94% to delineate species within Gardnerella, based on a survey across all available genomes. As noted earlier, the widely accepted ANI threshold for species-level classification is 95%, and deviating from this standard may affect consistency in the established genomic relatedness index. Since the analysis included genomes beyond the established type strains, it could be particularly valuable to re-examine the interspecies ANI threshold using only the genomes of the recognized type strain of species. This would help clarify whether 94% or 95% is the more appropriate cutoff for species demarcation.

3. The authors identified 11 new species based on a 94% ANI threshold, along with several subspecies. While ANI values were reported by the authors to be co-linear with dDDH values, it would be helpful to use dDDH as the primary basis for subspecies delineation—or at least present the dDDH values alongside ANI—to allow evaluation of their congruency. Since dDDH values are generally preferred for distinguishing subspecies (https://doi.org/10.1186/1944-3277-9-2) including these values would strengthen the robustness of the classification.

4. In Fig. 2a,b, I recommend adjusting the minimum ANI value of the heatmap to 94% and 95%. This would make the clustering of species and subspecies more clearly visible in relation to these threshold values, and help highlight the distinctions more effectively.

5. The species picketii, piotii, leopoldii, and swidsinskii were previously validated as distinct species through a polyphasic approach and genome-based analyses. It would be helpful if the manuscript addresses how these earlier findings align—or contrast—with the current classification of these taxa as subspecies. Revisiting the ANI and dDDH thresholds in light of the established results could strengthen the argument and ensure consistency with prior taxonomic frameworks. Applying the universally accepted ANI and dDDH thresholds for species delineation would help preserve these established designations and avoid confusion in the naming and classification of Gardnerella.

Reviewer #3

(Remarks to the Author)
The manuscript by Bouzek and Zepeda-Rivera et al. proposes a major taxonomic reclassification of the genus Gardnerella based on ANI, dDDH and phylogenomic analysis of 312 whole genome sequences derived from the authors' culture collection and publicly available data. The authors further identified lineage-specific differences in predicted metabolic pathways, virulence factors and defence systems through a syntenic pangenome analysis. The study also reports the identification of a cryptic plasmid from Gardnerella and demonstrates its use in making a Gardnerella-E. coli shuttle vector, subsequently used to transform the type strain of G. vaginalis.

The manuscript combines three components that are related; however, the result is somewhat disjointed. The first two components would package well as a taxonomic "renovation" of Gardnerella. The third component describes an exciting discovery and the development of a genetic tool that will be useful to the field.

The title does not mention the novel contribution described in the manuscript – the isolation of a cryptic plasmid and its use

in building a shuttle vector for transformation of Gardnerella vaginalis.

The lack of focus is also evident in the statement of the objective (lines 48-50), which lacks specificity.

Changes to the Discussion are needed. The Discussion is largely limited to re-stating the results without relating them to vaginal ecology or BV.

Proposed reclassification of Gardnerella:
It is not surprising that as whole genome sequences accumulate, additional subdivisions within existing taxa emerge. It was surprising that given all the effort to define taxonomic boundaries that the authors only went as far as proposing provisional names and did not meet the requirements for valid publication of new taxa as defined in the International Code of Nomenclature of Prokaryotes including deposit of type strains into two culture collections. The authors have put so much work into a carefully curated set of data and a thorough analysis, so it seems a shame to leave it as "provisional".

Some of the taxon proposals are not well supported since they are based on <5 isolates. Although it is technically possible to validly publish a new taxon based on a single isolate, multiple isolates (preferably epidemiologically distinct) obviously strengthen the case. Lack of multiple isolates is why Vaneechouette et al. did not propose names for multiple "genome species" identified in their study. A search of available shotgun metagenomic sequence data from vaginal microbiomes might yield at least some in silico support for these taxa and should be considered by the authors.

Further comment on the Bifidobacterium/Gardnerella distinction is needed – especially as it relates to the GTDB classification and that their framework places Gardnerella within Bifidobacterium despite the obvious and numerous differences (genome size, G+C content, etc.). This is a long-standing issue, the solution for which is most likely to make the case that Bifidobacterium is multiple genera. While beyond the scope of this study, the authors should take the opportunity to comment on/recommend potential resolutions.

The effort of the authors to provide historical perspective on the taxonomy of Gardnerella is appreciated, however, there is some clarification needed. In the section starting on line 80:
- (line 90) The citation for the subgroup A-D system is incorrect and this system was not based on whole genome sequencing. This system was based on cpn60 barcode sequencing and introduced by Jayaprakash et al. (2012) (ref 36).
- (line 92) "Nearly simultaneously…" clades 1-4 were described by Ahmed et al. (2012) (ref 30, not 42). The reference to Balashov et al. 2014 is also not relevant here.
- (line 101) refers to prior studies that use the "Set A and Set B" terminology but only one study is cited (30). If there are other examples of this usage (not sure there are), they should be included
- Figure 1A is helpful but Figure 1B is confusing as presented. What is the basis for the lines connecting Piot/Benito biotypes with the cpn60 subgroup/WGS clade terms? Both Jayaprakash et al. and Ahmed et al. reported Piot biotypes in their studies but those results do not align with Figure 1B
- Figure 1B – in the cpn60 subgroup/WGS clade column what are "B3", "A2", etc. (no slashes?)

The authors report using a 94% ANI threshold for designating "species" but a description of the threshold/requirements for defining "subspecies" should be provided. Also, please explain the rationale for using 94%.

Lines 123-131. The suggested shorthand for all the proposed species and subspecies is not consistent with rules of nomenclature and should be removed. Use of the shorthand within the manuscript is acceptable if explained.

Pangenome analysis:
The authors present a report of genes encoding virulence factors and predicted metabolic capabilities across the Gardnerella lineages. The results confirm current understanding that there are not strictly "pathogenic" and "non-pathogenic" Gardnerella, that there is diversity in metabolic features and the distribution of defence mechanisms like restriction modification systems. The results presented are generated in silico with no phenotypic evidence to support the conclusions and no specific hypotheses are presented or tested. The Discussion does not relate these findings to ecology in the vaginal microbiome or to BV and current models of etiology.

Line 201-204 – Reference 51 does not mention NanH4. NanH4 was described by Pelayo et al. PNAS (2024) as being encoded by a gene found in a minority of isolates and importantly it had no signal peptide and no association with sialidase activity. Similarly NanH1 ("Sialidase A") is not associated with sialidase activity and is likely intracellular. Unfortunately, PCR for NanH1 was used for several years as evidence of "sialidase positive" Gardnerella isolates. If the point is to look at distribution of virulence factors, this analysis should be limited to NanH2 and NanH3.

Along this same avenue, the observation that Set A genomes showed an "enrichment for sialidases" (line 380) is somewhat misleading since sialidase *activity* is largely restricted to Gs piotii and absent from G. vaginalis. This reflects the distribution of NanH2 and NanH3.

Lines 207-208. "type I pullulanase" should be replaced with "type II α-amylase-pullulanase" as described in the reference cited (54)

Set B genomes are described on line 384 as enriched for "putative adhesion and virulence proteins". Please clarify as there is not mention of adhesion elsewhere and "virulence proteins" is vague.

Cryptic plasmid
This is most exciting and novel aspect of the manuscript although the results are under-described and the methods are not sufficiently complete to allow readers to reproduce the work. For example, the actual conditions used in the "optimized" transformation are not specifically described. Publication of a laboratory protocol in an open access repository would be a service to the field.

The sequences of the plasmids shown in Figure 6D must be provided.

The diagram of pDNF01199S-01 in Figure 6D does not match what is shown in extended data Figure 9 where a helicase and toxin-antitoxin system are annotated. This should be clarified in the text and figures.

Minor comments

The term "standardized" is used in several places with no definition, e.g. line 149 "standardized names". Suggest removing all of these modifiers.

The term "unified" is used in the opening paragraph of the Discussion (unified taxonomic framework, unified Gardnerella nomenclature) without definition. Suggest removing these modifiers.

Software and database version numbers should be provided throughout.

Reviewer #4

(Remarks to the Author)
I co-reviewed this manuscript with one of the reviewers who provided the listed reports. This is part of the Nature Communications initiative to facilitate training in peer review and to provide appropriate recognition for Early Career Researchers who co-review manuscripts.

Version 1:

Reviewer comments:

Reviewer #1

(Remarks to the Author)
The revised manuscript is improved and a very comprehensive and useful study.

Reviewer #2

(Remarks to the Author)
The authors have substantially addressed my previous concerns and incorporated my suggestions. The responses to my review comments were comprehensive and satisfactory. The manuscript has undergone significant improvement in both clarity and rigor, and the revisions have strengthened the overall impact of the work. I have no further concerns.

Reviewer #3

(Remarks to the Author)
Thanks to the authors for carefully considering and responding to the reviewer comments. The manuscript is much improved. I have only a couple of minor comments.

In response to my comment #3, please replace "infer" with "imply" in the revised text where it says "Although these interpretations are based on comparative genomic potential and do not directly *infer* in vivo activity or clinical outcomes..."

Regarding Figure 1B. While I appreciate the effort to clarify the relationships between the Piot Biotypes and the cpn60 subgroup/Ahmed clade terminology, it remains true that these relationships are not clear. The number of isolates for which both Piot and sequence-based classifications are available is relatively small and (not surprisingly given they measure different things) they are not concordant. I'm not sure what "illustrative rather than deterministic" means, but the best solution might be to simply remove the arrows and say that there is insufficient data to define these relationships.

Comment #9. Regarding abbreviations. Explaining the use of these within this paper is fine, but I recommend not suggesting any use beyond this paper. It is always better to spell out organism names and with the exception of figure labels on occasion it is not necessary to abbreviate.

Reviewer #4

(Remarks to the Author)
I co-reviewed this manuscript with one of the reviewers who provided the listed reports. This is part of the Nature Communications initiative to facilitate training in peer review and to provide appropriate recognition for Early Career

Researchers who co-review manuscripts.

Open Access This Peer Review File is licensed under a Creative Commons Attribution 4.0 International License, which permits use, sharing, adaptation, distribution and reproduction in any medium or format, as long as you give appropriate credit to the original author(s) and the source, provide a link to the Creative Commons license, and indicate if changes were made.
In cases where reviewers are anonymous, credit should be given to 'Anonymous Referee' and the source.
The images or other third party material in this Peer Review File are included in the article's Creative Commons license, unless indicated otherwise in a credit line to the material. If material is not included in the article's Creative Commons license and your intended use is not permitted by statutory regulation or exceeds the permitted use, you will need to obtain permission directly from the copyright holder.
To view a copy of this license, visit https://creativecommons.org/licenses/by/4.0/

We thank the editorial team and reviewers for their careful evaluation of our manuscript. We appreciate the constructive comments and have revised the manuscript to address each point raised. Below, we respond to each comment in detail, with changes indicated by page and line numbers in the revised manuscript.

Reviewer 1

Comment #1

This study is a tour-de-force analysis of Gardnerella genomics and will be highly useful to the field.

Response :

We thank the reviewer for their positive comments and for recognizing the potential value of this work to the field.

Comment #2

The paragraph describing the evolution of the naming conventions for Gardnerella (lines 80-102) is overwrought. The recognition that Gardnerella are multiple different species has been clarifying for the field and has allowed it to move on from Ahmed's clade designations to actual species names and the ability to characterize those species. Thus, the authors should tone-down their description of the confusion brought about by the multiple attempts to accurately describe the diversity of Gardnerella. In particular, the claim that the designation of species is compounding the complexity (line 100) should be removed, especially since the authors in this study are designating new species in the Gardnerella genus.

Response :

We agree that this section was overly long. Our intention was to provide historical context rather than to criticize prior classification schemes. The paragraph is now more succinct.

The original text read:

Given the importance of Gardnerella species diversity in BV, we first identified the number of lineages present in our genome collection. Although different Gardnerella species have been assigned through various approaches, the naming of these groups has been a consistent challenge for the Gardnerella field despite extensive and laudable efforts from multiple research groups (Fig. 1). In summary, the earliest Gardnerella subtyping method came from Piot et al. where Gardnerella biotypes were based on sugar hydrolysis phenotypes. When this approach proved insufficient to capture the diversity of the genus, it was expanded to 17 biotypes by adding three fermentation targets[27]. The use of early genomic methods included restriction fragment length polymorphism (RFLP) and

amplified ribosomal DNA restriction analysis (ARDRA), which identified distinct Gardnerella genotypes. Whole-genome comparisons brought about another naming scheme seeking to encompass all strains, where "A", "B", "C", and "D" were proposed designations for a newer condensed species assignment. Nearly simultaneously, another method was proposed, using "1", "2", "3", and "4" for the same clades, which led to a disjointed hybrid designation (C/1, B/2, A/4, and D/3). The species were then renamed as reassorted ecotypes, using yet another nomenclature, one that incorporated the genomic context of the distinct types. After whole-genome sequencing became widely available, three more competing standards based on ANI emerged, describing thirteen genomic species (Gsp), nine genomospecies (GS), or nine different genotypes (GGtypes). Formal naming of a limited subset of these species, included G. vaginalis, G. pickettii, G. piotii, G. leopoldii, G. swidsinskii, and G. greenwoodii, and has further compounded this complex nomenclature (Figure 1). Despite the use of different classification schemes, prior studies consistently identified two distinct Gardnerella subgenera (Set A and Set B), each encompassing specific lineages.

The revised text now reads (lines 83-101):

Given the importance of *Gardnerella* species diversity in BV[1], we first assessed the number of lineages present in our genome collection. *Gardnerella* has been classified using various approaches[2-7], but consistent naming has been a challenge for *Gardnerella* despite extensive efforts[4,8-10] (Fig. 1). Early subtyping by Piot *et al*.[8] relied on sugar hydrolysis phenotypes and later expanded to 17 biotypes[9]. Genomic methods such as restriction fragment length polymorphism (RFLP)[10] and amplified ribosomal DNA restriction analysis (ARDRA)[11] identified several distinct genotypes. PCR and whole-genome sequence comparisons introduced two new naming schemes including designations "A - D"[12] or "1 - 4"[2] which led to hybrid labels ("C/1", "B/2", "A/4", and "D/3")[13]. These species were then reframed as reassorted ecotypes[5] incorporating the genomic context of the distinct types. With widespread whole-genome sequencing, additional standards based on ANI emerged, describing thirteen genomic species (Gsp)[4], nine genomospecies (GS)[6], or nine different genotypes (GGtypes)[14]. Subsequent formal naming of a subset of these species included *G. vaginalis*[15], *G. pickettii*[16], *G. piotii*[4], *G. leopoldii*[4], *G. swidsinskii*[4], *G. greenwoodii*[16], *G. lacydonensis*[17], *G. bretellae*[17], *G. massiliensis*[17], and *G. phocaeensis*[17] (Fig. 1). Despite the use of diverse classification schemes, phylogenetic analyses across studies consistently recover a deep bifurcation within *Gardnerella*, with the same two major lineages forming the basal split of the genus. While this division has been explicitly designated as "Set A" and "Set B" only by Ahmed *et al*., analogous groupings confirm the primary branching structure in multiple independent phylogenetic frameworks[2,4-7,12,14,18-20].

Comment #3

The taxonomy rules for naming prokaryotic species are somewhat complex, but I do not think the current accepted names for Gardnerella species can simply be discarded as proposed in lines 121-125 and Fig 1 and Fig 2. I refer the authors to the International Code of Nomenclature of Prokaryotes. Prokaryotic Code (2022 Revision) (doi.org/10.1099/ijsem.0.005585). In particular, on the subject of priority, I think the names for three of the species will have to be retained (Chapter 3, Section 5, Rule 23a). The authors propose the species G. aggregata, but it contains the named species G. leopoldii and G. swidsinskii. According to the Code, the authors can choose one of those to be the new species name. Similarly, the proposed G. sialicivorans would need to be either G. piottii or G. pickettii. The proposed new species G. media would need to be G. greenwoodii. Also, I would encourage the authors not to choose "media" as a species name since searches for "Gardnerella media" are likely to return lots of hits for NYCIII and HBT plates and not the desired information about the bacteria.

Response:

The reviewer is correct, and we thank them for drawing our attention to Rule 23a of the International Code of Nomenclature of Prokaryotes and for the detailed guidance on priority. During editing and resubmission of this manuscript, an additional publication by Allini Ntiguemassa *et al*. validly named four *Gardnerella* lineages. As these were validly published within the IJSEM journal, and take precedent, we have updated our nomenclature to include these names.

Specifically, *G. piotii* precedes *G. pickettii* so these become *G. piotii* subspecies *piotii* and *G. piotii* subspecies *pickettii*. The final subspecies of *G. piotii* may retain the subspecies name *sialicivorans* as *G. piotii* subspecies *sialicivorans*. As *G. leopoldii* and *G. swidsinskii* were both validly published on the same date and manuscript, we selected *G. leopoldii* to represent the new species since Leopold's work in *Gardnerella* precedes that of Swidsinski. The newly named *Gardnerella phocaeensis* is a subspecies of *Gardnerella leopoldii* and will become *Gardnerella leopoldii* subspecies *phocaeensis.*

Following along this revision, *G. media* is now *G. greenwoodii* with the type strain set within *G. greenwoodii* subspecies *greenwoodii*. The other two subspecies are now *G. greenwoodii* subspecies *intermedia* and *G. greenwoodii* subspecies *bretellae*. The final newly named *Gardnerella lacydonensis* is in fact a subspecies of *Gardnerella vaginalis* and shall become *Gardnerella vaginalis* subspecies *lacydonensis*.

Taking this opportunity to revise names for clarity and after consultation with Dr. Marko Kostovski, an expert in nomenclature and now co-author, we will now use *G. hutchinsoni* for the former *G. coleohominis* (Gsp13) as the correct Greek translation would be *G. koleohominis* and that would interfere with our two-letter naming scheme.

Figure 1 is updated to show these changes.

REDACTED

Comment #4

The claim that "variable lineage-dependent pathogenic potential exists across Gardnerella" (lines 211-212) is an over-interpretation of the data presented. The statement implies that all the species and subspecies are or can be pathogens, and the bioinformatic analysis is not sufficient to make that claim. Perhaps the authors meant something else and just need to state that more specifically.

Response to reviewer:

We agree that the statement was ambiguous and over-reaching. We have revised to highlight lineage-dependent variation in metabolic capacity and the presence of putative virulence-associated genes without making claims about pathogenicity.

The text originally read:

Our results support the hypothesis that variable lineage-dependent pathogenic potential exists across Gardnerella.

The text now reads (lines 227-231):

Comparisons between *Gardnerella* Set A and Set B showed that the four sialidase genes (*nanH1-nanH4*) are significantly enriched in Set A genomes, while vaginolysin (*vly*) is significantly enriched in Set B genomes (Fig. 4e, Extended Data Fig. 5), highlighting lineage-dependent differences in metabolic capacity and the distribution of putative virulence-associated genes across *Gardnerella*.

Comment #5

The identification of a new phage in Gardnerella (phage gamma) is exciting (line 257). The authors should include a figure showing the phage gamma gene map from a representative strain.

Response to reviewer:

> We agree that this is important and missing information. We have now included the phage map for vB_Gva_AB1 and the phage map for a representative pGamma as Extended Figures 6a and 6b.
>
> We have also amended the title to "*A Syntenic Pangenome of Gardnerella Reveals Novel Plasmids and Phage, Taxonomic Boundaries, and Species-Level Stratification of Metabolic and Virulence Potential*", as we agree with the reviewer they be noticed by the broader interest to the field if the title reflects this finding.

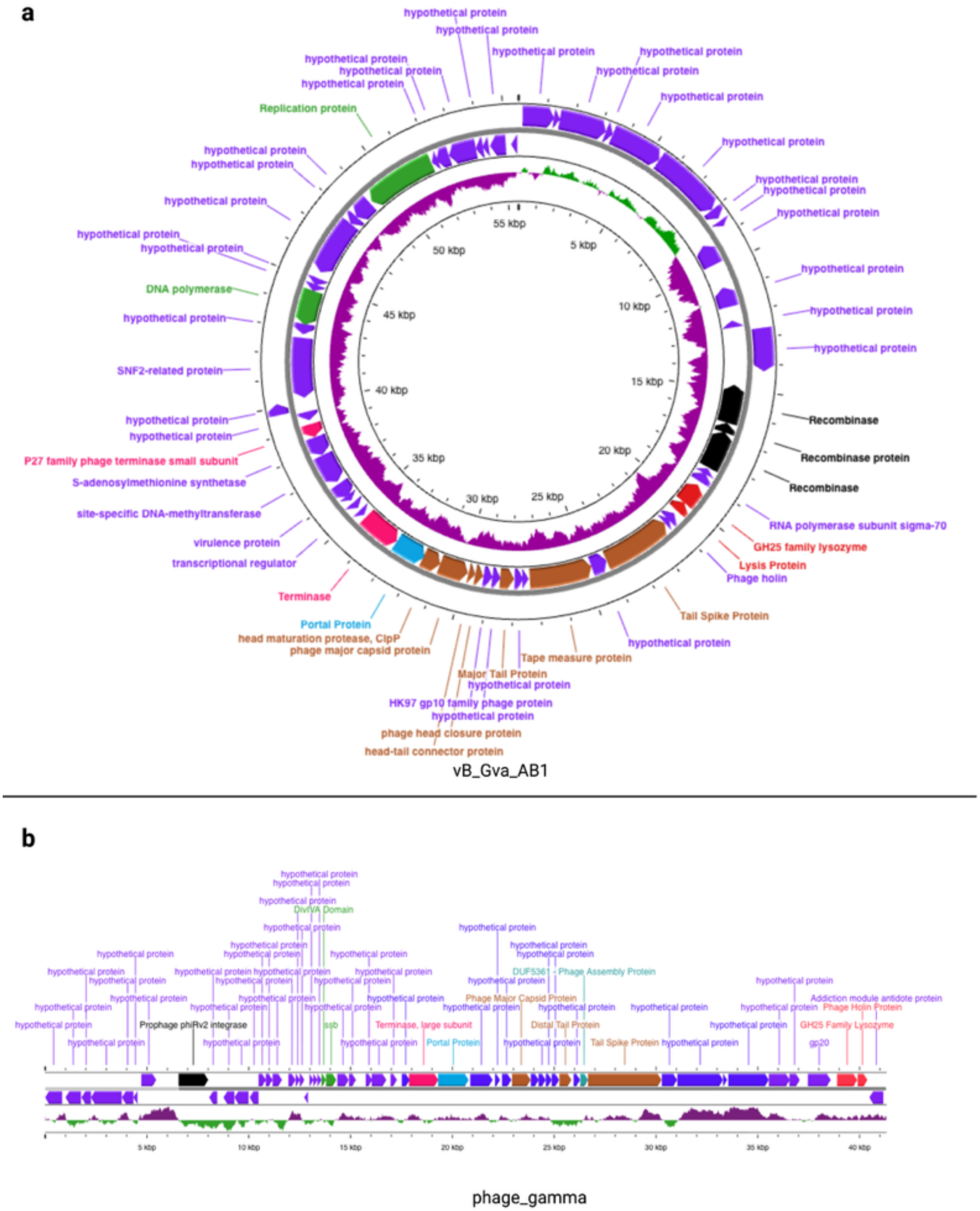

a
hypothetical protein
Replication protein
DNA polymerase
SNF2-related protein
P27 family phage terminase small subunit
S-adenosylmethionine synthetase
site-specific DNA-methyltransferase
virulence protein
transcriptional regulator
Terminase
Portal Protein
head maturation protease, ClpP
phage major capsid protein
Major Tail Protein
HK97 gp10 family phage protein
phage head closure protein
head-tail connector protein
Tape measure protein
Tail Spike Protein
Phage holin
Lysis Protein
GH25 family lysozyme
RNA polymerase subunit sigma-70
Recombinase
Recombinase protein
55 kbp
5 kbp
10 kbp
15 kbp
20 kbp
25 kbp
30 kbp
35 kbp
40 kbp
45 kbp
50 kbp
vB_Gva_AB1
b
phage_gamma

Minor Comments

1. Line 72. Correct spelling to "Scardovia".

Line changed from "…and closely related *Cardovia*…" to "…and closely related scardovial genera…"

2. Contrary to what is stated in the text (line 105), Extended data Fig. 2 does not indicate which species are Set A and Set B.

Thank you for highlighting this omission. The figure has been changed to show Set A and Set B.

Original Figure:

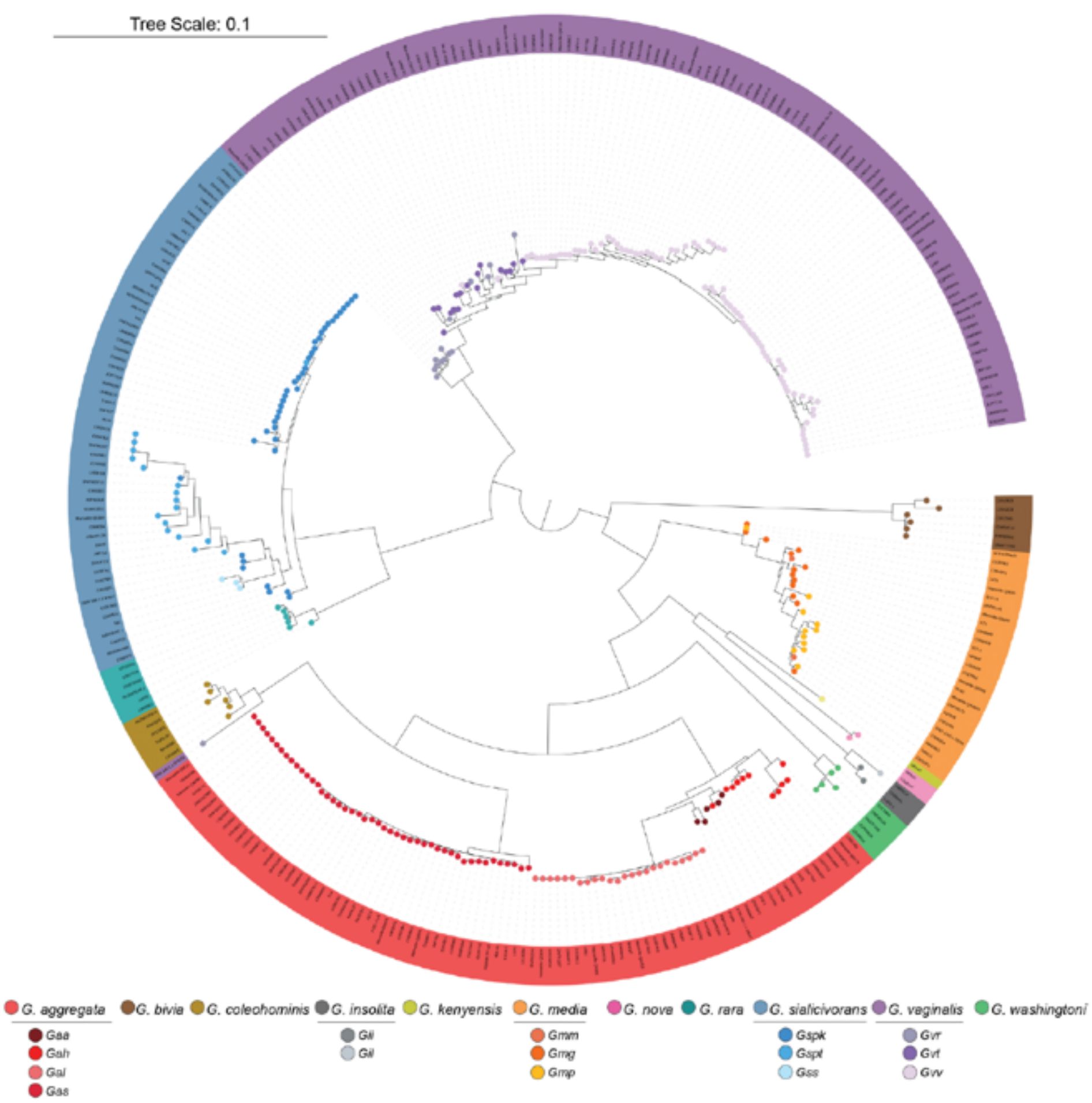

Extended Data Fig. 2: *Gardnerella cpn60* gene sequence dendrogram

Maximum-likelihood dendrogram based on MUSCLE-aligned *cpn60* gene sequences from *Gardnerella* genomes. Label indicates strain identifier, while label and tip point color represent lineage.

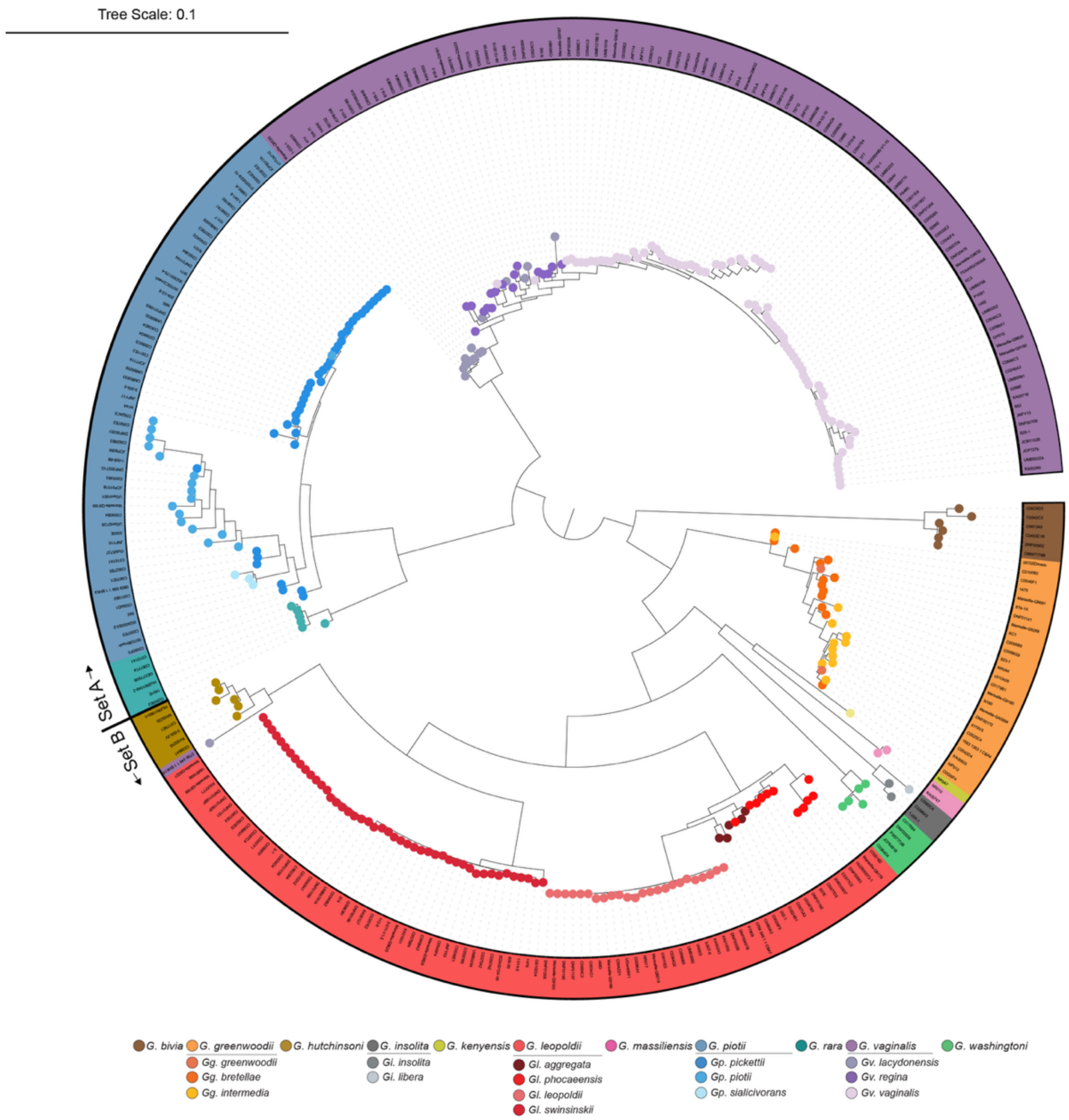

3. The presence of tetM in Gardnerella providing tetracycline resistance was published and should be cited in line 330. Roberts MC et al. 1986 (doi: 10.1128/AAC.30.5.810).

We thank the reviewer for highlighting this omission. The above citation is now included.

4. Only report significant figures for the transformation efficiency (line 346).

The line *"…from a minimum of $2.30x10^0$ transformants per µg of DNA to $6.85x10^2$"* was altered to remove the scientific notation. "from a minimum of 2.3 transformants per µg of DNA up to 685 per µg".

Reviewer 2

Comment #1

The classification of Gardnerella within the family Bifidobacteriaceae remains unresolved. Multiple phylogenetic analyses have consistently positioned members of this genus within Bifidobacterium (https://doi.org/10.1128/AEM.02188-20,

https://doi.org/10.1111/eva.12555, https://doi.org/10.1186/s12864-017-3955-4), suggesting that reclassification may be warranted. This proposed reclassification is reflected in the GTDB (as acknowledged in your manuscript). However, no formal taxonomic proposals have yet been made, and the genus name Gardnerella continues to be valid and recognized in current nomenclature.

In lines 77–79, the authors state that Gardnerella and Bifidobacterium are distinct genera, basing this claim on differences in G+C content, genome length, and—surprisingly—an ANI threshold of 73–75%. Is the ANI threshold for genus-level demarcation universally accepted? To date, ANI thresholds have only been formally established for species-level delineation (95%). Regarding the presented heatmap of ANI values (Extended Data Fig. 1c), it is unclear whether the clustering was derived from phylogenetic placements or hierarchical clustering of ANI values. To strengthen the analysis, I recommend including a genome-based phylogenetic tree of all available type strains within Bifidobacteriaceae. This would allow verification of the phylogenetic placement of Gardnerella species and clarify their distinction from Bifidobacterium.

Response to reviewer:

We thank the reviewer for this detailed comment. We agree that the placement of *Gardnerella* within the family *Bifidobacteriaceae* is debated and that multiple genome-based analyses, including GTDB, currently position *Gardnerella* within or closely allied to *Bifidobacterium*. This manuscript is intended as the genome-based phase of a broader taxonomic re-evaluation of *Gardnerella*, rather than as a final formal proposal to merge or synonymize genera. Accordingly, we retain the genus name *Gardnerella* in the present work, consistent with current nomenclature, while explicitly presenting our analyses as contributing additional evidence toward future reclassification.

We also agree with the reviewer that no universally accepted ANI threshold exists for genus-level demarcation, and that ANI thresholds are formally established only for species-level delineation (~95%). Our intention was not to imply the existence of a formal genus-level ANI standard. Rather, our use of ANI follows the same conceptual framework originally applied to establish species-level thresholds - namely, identifying natural discontinuities (“ANI gaps”) in pairwise similarity distributions, rather than applying an *a*

priori cutoff. We have revised the text to clarify this distinction and to frame ANI as an empirical comparative metric used to contextualize genus-level relationships, rather than as a formal criterion. In addition, we have included more formal separation metrics, including Amino Acid Identity (AAI), which were not included previously (Extended Data Figure 1a-f).

Regarding Extended Data Fig. 1c, the clustering was based on hierarchical clustering of pairwise ANI values, not phylogenetic inference; this has now been clarified in the figure legend. In response to the reviewer's suggestion, we have additionally included a genome-based phylogenetic tree of type strains within *Bifidobacteriaceae*, including *Gardnerella* and *Bifidobacterium*. This phylogeny supports the close evolutionary relationship between these genera while maintaining coherent clustering of *Gardnerella* lineages and is presented as Extended Figure 1d.

We have revised the manuscript throughout to clarify that this study represents a stepwise genomic framework for reclassification, rather than a complete or final taxonomic resolution.

Extended Data Fig. 1 originally appeared as this combined figure:

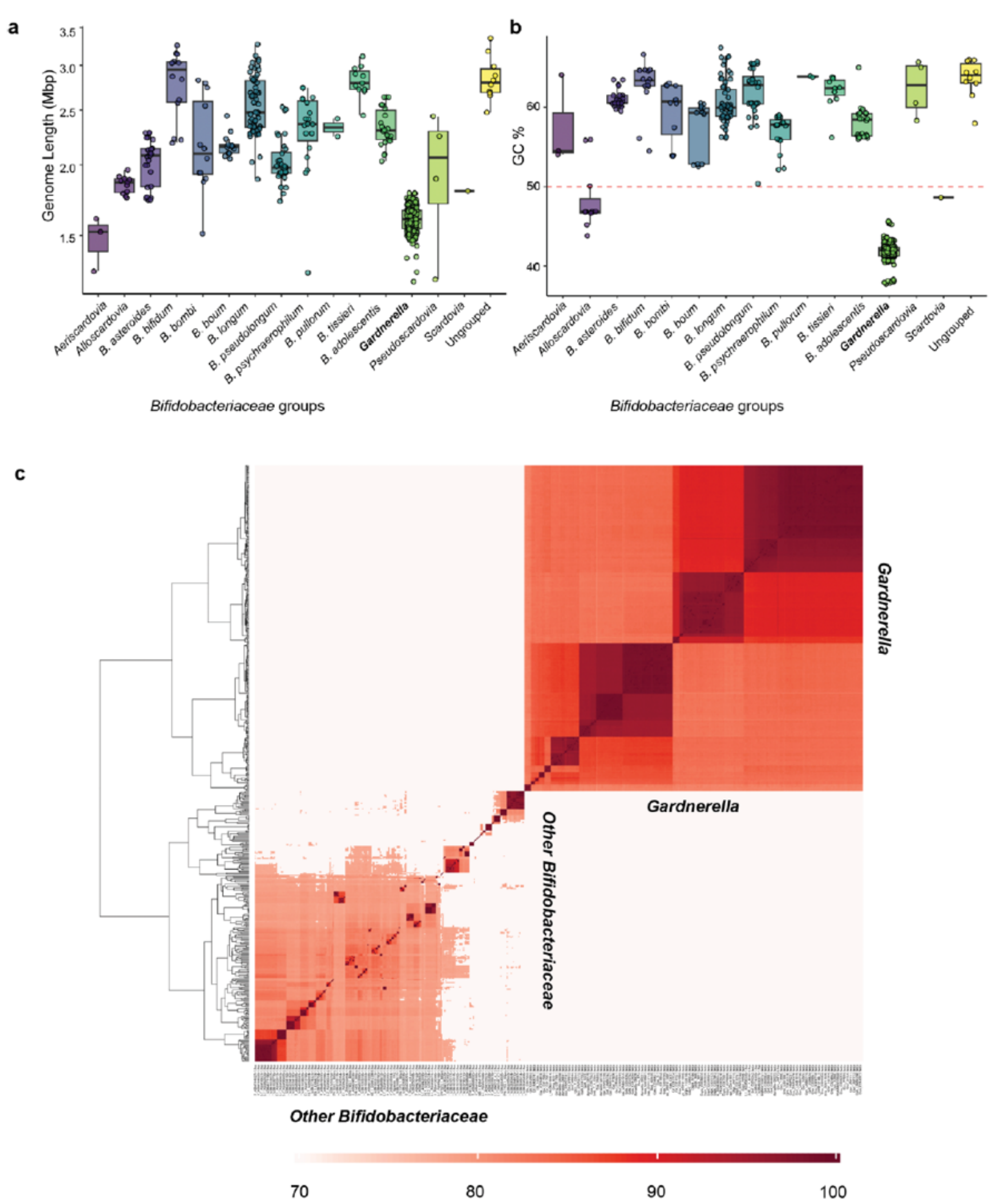

The original caption read:

> ***Extended Data Fig. 1: Comparisons between Bifidobacterium, Gardnerella, and other closely related genera***
> *Comparisons of **a**, genome length and **b**, G+C percentage of genomes classified as subfamilies within Bifidobacteriaceae. **c**, Average nucleotide identity (ANI) value comparisons between Bifidobacteriaceae genomes. Key indicates corresponding values to each color.*

The new figures now appear as:

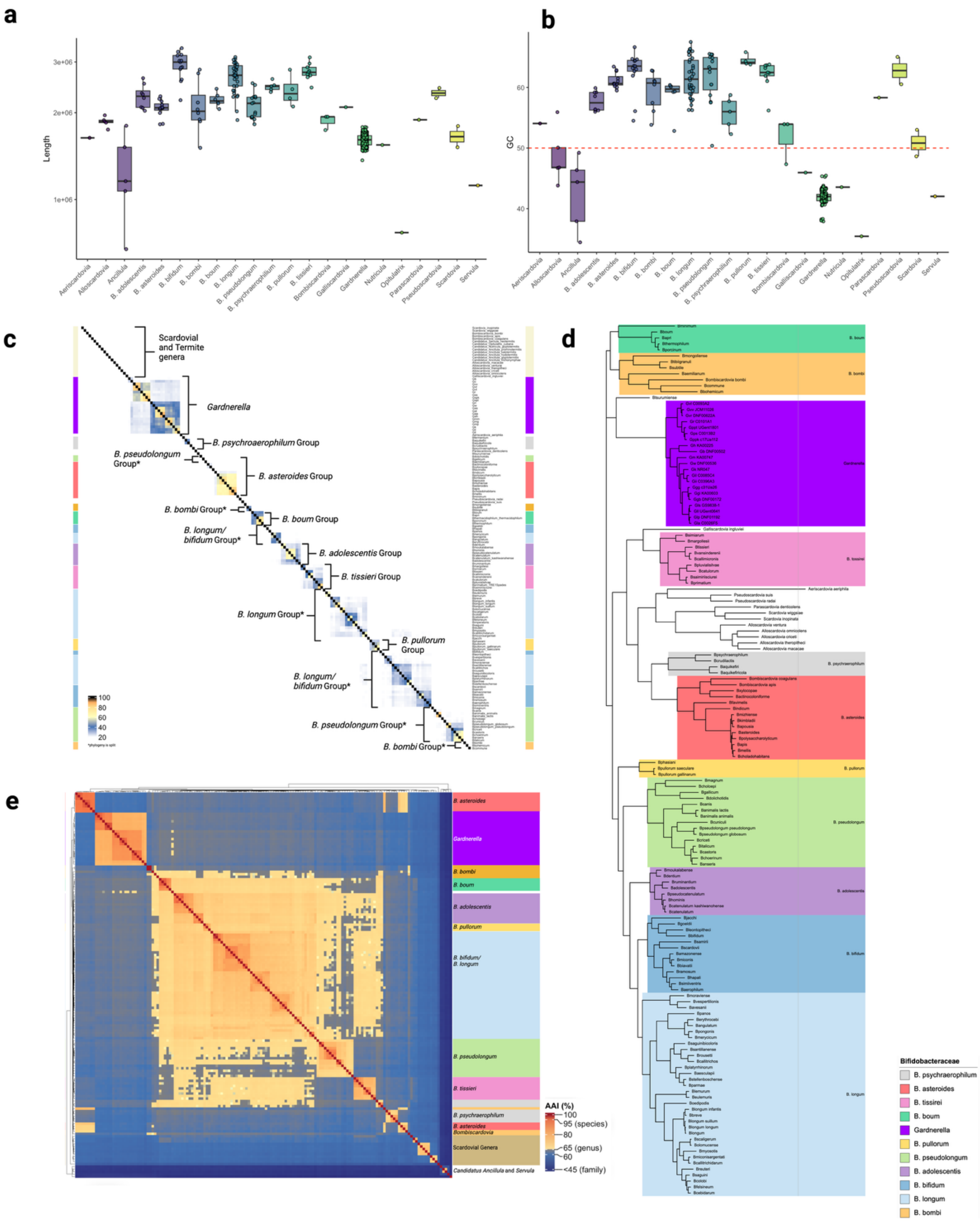

a
3e+06
2e+06
1e+06
Length
Aeriscardovia
Alloscardovia
Ancillula
B. adolescentis
B. asteroides
B. bifidum
B. bombi
B. boum
B. longum
B. pseudolongum
B. psychraerophilum
B. pullorum
B. tissieri
Bombiscardovia
Galliscardovia
Gardnerella
Nutriculla
Oplitulatrix
Parascardovia
Pseudoscardovia
Scardovia
Servula
b
60
50
40
GC
Aeriscardovia
Alloscardovia
Ancillula
B. adolescentis
B. asteroides
B. bifidum
B. bombi
B. boum
B. longum
B. pseudolongum
B. psychraerophilum
B. pullorum
B. tissieri
Bombiscardovia
Galliscardovia
Gardnerella
Nutriculla
Oplitulatrix
Parascardovia
Pseudoscardovia
Scardovia
Servula
c
Scardovial and Termite genera
Gardnerella
B. psychroaerophilum Group
B. pseudolongum Group*
B. asteroides Group
B. bombi Group*
B. boum Group
B. longum/ bifidum Group*
B. adolescentis Group
B. tissieri Group
B. longum Group*
B. pullorum Group
B. longum/ bifidum Group*
B. pseudolongum Group*
B. bombi Group*
100
80
60
40
20
*phylogeny is split
d
B. boum
B. bombi
Gardnerella
B. tissieri
Aeriscardovia aeriphila
B. psychraerophilum
B. asteroides
B. pullorum
B. pseudolongum
B. adolescentis
B. bifidum
B. longum
Bifidobacteraceae
B. psychraerophilum
B. asteroides
B. tissierei
B. boum
Gardnerella
B. pullorum
B. pseudolongum
B. adolescentis
B. bifidum
B. longum
B. bombi
e
B. asteroides
Gardnerella
B. bombi
B. boum
B. adolescentis
B. pullorum
B. bifidum/ B. longum
B. pseudolongum
B. tissieri
B. psychraerophilum
B. asteroides
Bombiscardovia
Scardovial Genera
Candidatus Ancillula and Servula
AAI (%)
100
95 (species)
80
65 (genus)
60
<45 (family)

The new caption reads:

Extended Data Fig. 1: Genomic comparisons among members of the family *Bifidobacteriaceae*
Comparative genomic analyses across representative genera of the family *Bifidobacteriaceae*, including subgroups of *Bifidobacterium* (as defined by Alessandri *et al.*), the "Scardovial" genera (*Aeriscardovia*, *Alloscardovia*, *Bombiscardovia*, *Galloscardovia*, etc.), and genera associated with termite gut microbiota (*Ancillula*, *Servulla*, etc.).

a, Genome length comparisons across representative genera. Relative to *Bifidobacterium*, genomes of *Gardnerella* (Kruskal–Wallis with Bonferroni correction, $p < 0.001$) and *Alloscardovia* ($p = 0.052$) are smaller. *Gardnerella* genomes are also significantly smaller than those of *Alloscardovia* ($p = 0.009$). Other comparisons involving Scardovial genera lack sufficient representation or exhibit broad variance that limits statistical inference. **b,** Genomic G+C content across selected genera. *Gardnerella*, *Ancillula*, and *Alloscardovia* exhibit significantly lower G+C percentages than *Bifidobacterium* ($p < 0.001$, $p = 0.0113$, and $p = 0.0179$, respectively).

c, Digital DNA–DNA hybridization (dDDH) values among representative genomes. The species-level threshold (>70%) is highlighted in yellow; intermediate similarity (~40%) is shown in blue, and low similarity (<20%) in white. *Gardnerella* forms a monophyletic cluster within *Bifidobacteriaceae*. Groups marked with an asterisk indicate polyphyletic lineages. Colors along the axes correspond to the *Bifidobacterium* groups defined by Alessandri *et al.*

d, Conserved protein phylogeny of *Bifidobacteriaceae* type strains. Persistent PPanGGOLiN gene clusters were extracted and used to construct a neighbor-joining tree. *Gardnerella* forms a monophyletic lineage within the broader *Bifidobacteriaceae*, with the nearest cluster including a member of *Bombiscardovia*.

e, Amino acid identity (AAI) among type strains. Pairwise AAI values were calculated and visualized as a heatmap. Color gradients correspond to commonly applied genomic thresholds: dark blue (<45%, typical of inter-family divergence), light blue (45–65%, inter-generic range), yellow (>65%, genus-level coherence), and red (~100%, near-identical comparisons).

f, PPanGGOLiN tile map of the pangenome derived from *Bifidobacteriaceae* type strains. Nodes (rows) correspond to gene clusters within the persistent fraction of the pangenome (orange bar). Shell partitions are indicated by green shades. The accompanying dendrogram is derived from a similarity matrix of gene presence–absence patterns. *Gardnerella* clusters with the Scardovial genera. The tile plot shows the relatively few persistent genes are uniquely shared between *Bifidobacterium* and *Gardnerella*.

Comment #2

The authors applied an ANI value of 94% to delineate species within Gardnerella, based on a survey across all available genomes. As noted earlier, the widely accepted ANI threshold for species-level classification is 95%, and deviating from this standard may affect consistency in the established genomic relatedness index. Since the analysis included genomes beyond the

established type strains, it could be particularly valuable to re-examine the interspecies ANI threshold using only the genomes of the recognized type strain of species. This would help clarify whether 94% or 95% is the more appropriate cutoff for species demarcation.

<u>Response to reviewer</u>:

> We agree that the choice of 94% warrants careful justification, and we appreciate the reviewer's suggestion on how to best handle this nuance. To clarify the impact of this threshold on our classification, we have added the suggested visualization (below) as Extended Data Figure 3e. This heatmap shows that for the verified type strains and those we choose for these species, if the ANI values are averaged over the Query-Subject pairs the ANI values are over the threshold of 95%. Reviewer #3 also raised this issue in Comment #8, and we provide a more detailed discussion of the rationale and supporting analyses in our response below.

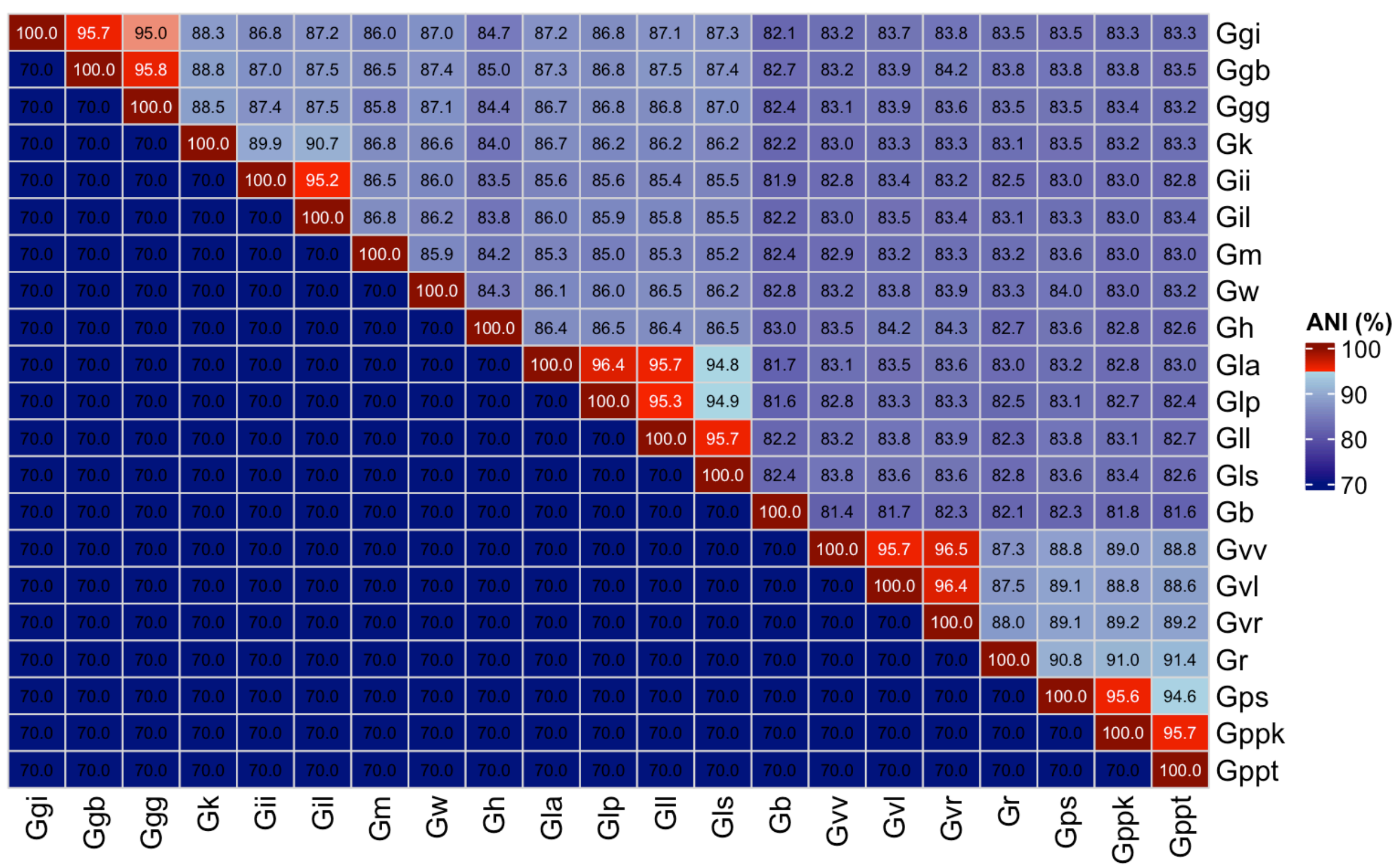

Extended Figure 3e - ANI of the type strains of *Gardnerella* species and subspecies. The mean of the pairwise ANI (i,j) and (j,i) represented as just the upper triangle. Values from 100% to 95% are colored in increasingly lighter shades of red, values below 95.0% are colored in increasingly darker shades of blue.

Comment #3

The authors identified 11 new species based on a 94% ANI threshold, along with several subspecies. While ANI values were reported by the authors to be co-linear with dDDH values, it would be helpful to use dDDH as the primary basis for subspecies delineation—or at least present the dDDH values alongside ANI—to allow evaluation of their congruency. Since dDDH values are generally preferred for distinguishing subspecies (https://doi.org/10.1186/1944-3277-9-2) including these values would strengthen the robustness of the classification.

Response to reviewer:

We thank the reviewer for this insightful suggestion. We agree that presenting dDDH data alongside ANI data strengthens the robustness of the subspecies delineation. We have now added these data as a heatmap as Extended Data Figure 3a, also as an addition to Supplementary Table 4, with dDDH values for all relevant lineages and highlighted their congruence with ANI-based assignments. We also note that in the rare cases that the ANI does fall outside the 95% boundary that the reciprocal dDDH Formula 1 boundary is sufficiently above its threshold of 70%.

Extended Figure 3a:

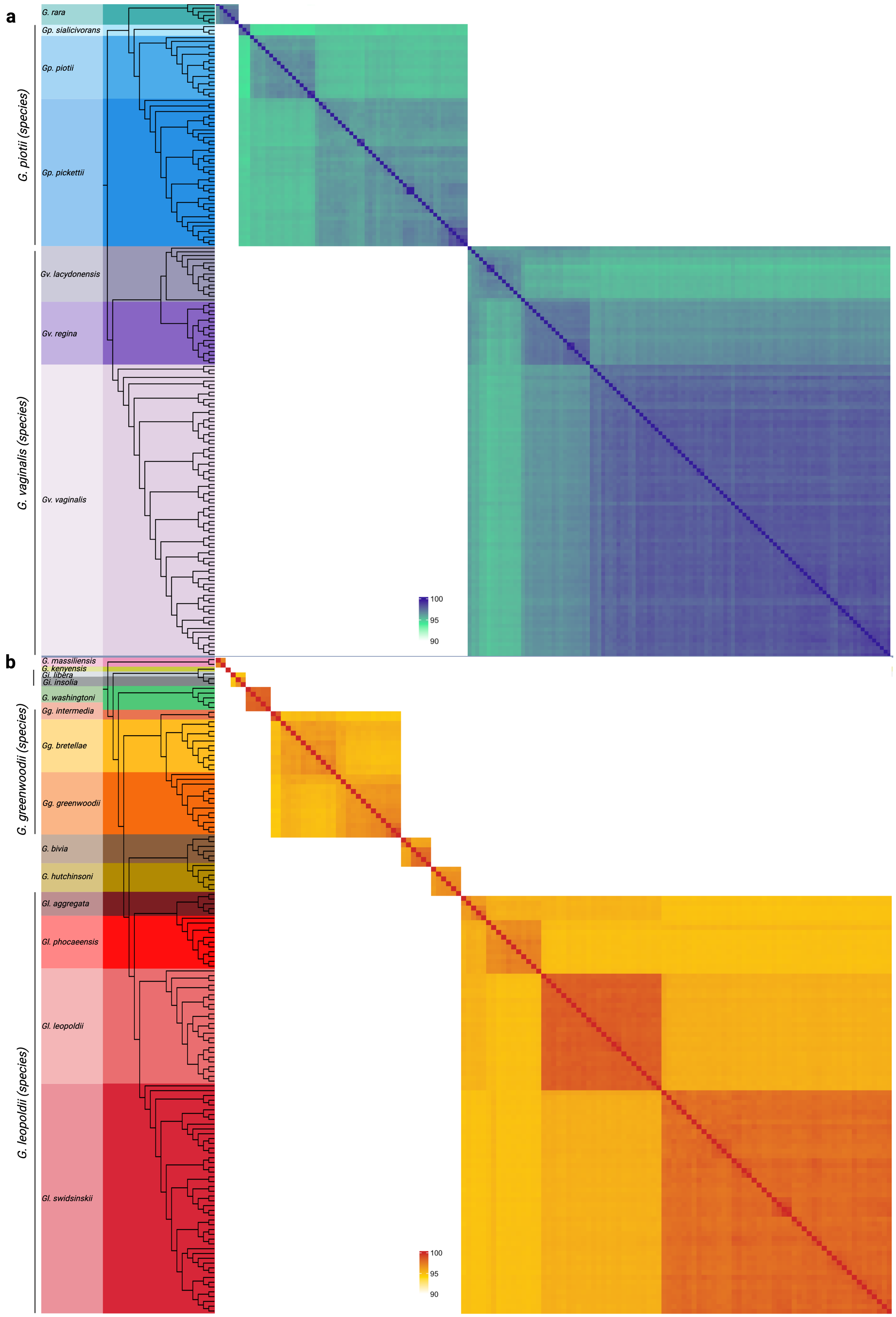

a
G. rara
Gp. sialicivorans
Gp. piotii
Gp. pickettii
G. piotii (species)
Gv. lacydonensis
Gv. regina
Gv. vaginalis
G. vaginalis (species)
100
95
90
b
G. massiliensis
G. kenyensis
Gl. libera
Gl. insolia
G. washingtoni
Gg. intermedia
Gg. bretellae
Gg. greenwoodii
G. greenwoodii (species)
G. bivia
G. hutchinsoni
Gl. aggregata
Gl. phocaeensis
Gl. leopoldii
Gl. swidsinskii
G. leopoldii (species)
100
95
90

Data added to Extended Table 4:

Query Species	Query Subspecies	Subject Species	Subject Subspecies	ANI [range]	dDDH Formula 1 [range]	dDDH Formula 3 [range]
G. bivia		*G. bivia*		98.7 [98.7, 98.7]	95 [95, 95]	96 [96, 95.5]
G. greenwoodii	*bretellae*	*G. greenwoodii*	*bretellae*	97.2 [96.7, 97.6]	89.8 [80.5, 95.5]	89 [82, 94.5]
G. greenwoodii	*bretellae*	*G. greenwoodii*	*greenwoodii*	95.5 [95, 96]	87.5 [78.5, 91.8]	85 [77, 88.8]
G. greenwoodii	*bretellae*	*G. greenwoodii*	*intermedia*	95.6 [95.4, 95.9]	83.3 [73, 88.1]	81 [73, 84.8]
G. greenwoodii	*greenwoodii*	*G. greenwoodii*	*bretellae*	95.5 [95, 95.9]	87.5 [78.5, 91.8]	85 [77, 88.8]
G. greenwoodii	*greenwoodii*	*G. greenwoodii*	*greenwoodii*	97.3 [97.1, 97.6]	90.4 [87.4, 93.2]	90 [87, 92.7]
G. greenwoodii	*greenwoodii*	*G. greenwoodii*	*intermedia*	94.8 [94.6, 95.1]	84.8 [83, 86.1]	81 [80, 82.3]
G. greenwoodii	*intermedia*	*G. greenwoodii*	*bretellae*	95.6 [95.3, 95.8]	83.3 [73, 88.1]	81 [73, 84.8]
G. greenwoodii	*intermedia*	*G. greenwoodii*	*greenwoodii*	94.8 [94.7, 95.1]	84.8 [83, 86.1]	81 [80, 82.3]
G. greenwoodii	*intermedia*	*G. greenwoodii*	*intermedia*	99.2 [99, 99.3]	99 [98.7, 99.3]	99 [99, 99.4]
G. hutchinsoni		*G. hutchinsoni*		98 [98, 98]	93.1 [93.1, 93.1]	93 [93, 92.8]
G. leopoldii	*aggregata*	*G. leopoldii*	*leopoldii*	95.9 [95.6, 96.1]	85.6 [74.6, 91.4]	84 [74, 88.7]
G. leopoldii	*aggregata*	*G. leopoldii*	*phocaeensis*	96.3 [96.3, 96.3]	85 [84.5, 85.5]	84 [83, 84.3]
G. leopoldii	*aggregata*	*G. leopoldii*	*swidsinskii*	95 [94.9, 95.2]	75.6 [70.4, 80.2]	74 [70, 78.2]
G. leopoldii	*leopoldii*	*G. leopoldii*	*aggregata*	95.9 [95.5, 96]	85.6 [74.6, 91.4]	84 [74, 88.7]
G. leopoldii	*leopoldii*	*G. leopoldii*	*leopoldii*	98.8 [98.4, 99.2]	91.1 [78.8, 97.8]	93 [83, 98]
G. leopoldii	*leopoldii*	*G. leopoldii*	*phocaeensis*	95.2 [94.8, 95.4]	77.7 [68, 82.3]	76 [68, 80.2]
G. leopoldii	*phocaeensis*	*G. leopoldii*	*swidsinskii*	94.9 [94.8, 95.2]	72.9 [68.2, 77.9]	72 [68, 76.1]
G. leopoldii	*swidsinskii*	*G. leopoldii*	*aggregata*	95 [94.8, 95.2]	75.6 [70.4, 80.2]	74 [70, 78.2]
G. leopoldii	*swidsinskii*	*G. leopoldii*	*leopoldii*	95.8 [95.3, 96.2]	74.2 [60.7, 80.5]	74 [63, 80.4]
G. leopoldii	*swidsinskii*	*G. leopoldii*	*phocaeensis*	94.9 [94.7, 95.1]	72.9 [68.2, 77.9]	72 [68, 76.1]
G. leopoldii	*swidsinskii*	*G. leopoldii*	*swidsinskii*	98.4 [97.9, 99.1]	90.2 [82.5, 98.4]	92 [86, 98.5]
G. massiliensis		*G. massiliensis*		97.9 [97.9, 98]	90 [88.6, 92.1]	90 [89, 92]
G. piotii	*pickettii*	*G. piotii*	*pickettii*	97.3 [96.7, 98.9]	91.2 [84.5, 99.4]	90 [84, 99]
G. piotii	*pickettii*	*G. piotii*	*piotii*	95.9 [95.6, 96.4]	84.2 [78.4, 90.1]	82 [77, 87.7]
G. piotii	*piotii*	*G. piotii*	*pickettii*	95.9 [95.6, 96.4]	84.2 [78.4, 90.1]	82 [77, 87.7]
G. piotii	*piotii*	*G. piotii*	*piotii*	97.2 [96.8, 97.5]	89.2 [81.5, 93.3]	89 [82, 92.2]
G. vaginalis	*lacydonensis*	*G. vaginalis*	*lacydonensis*	97.1 [96.3, 97.6]	87.4 [82.1, 92.1]	87 [82, 91.4]
G. vaginalis	*lacydonensis*	*G. vaginalis*	*regina*	96.4 [95.9, 97.2]	84.4 [79.8, 87.9]	84 [79, 86.5]
G. vaginalis	*lacydonensis*	*G. vaginalis*	*vaginalis*	95.9 [95.4, 96.6]	82.1 [74.5, 88.2]	81 [75, 86.2]
G. vaginalis	*regina*	*G. vaginalis*	*lacydonensis*	96.4 [96, 97.2]	84.4 [79.8, 87.9]	84 [79, 86.5]
G. vaginalis	*regina*	*G. vaginalis*	*regina*	97.8 [97.8, 97.8]	90.9 [90.9, 90.9]	91 [91, 91.4]
G. vaginalis	*regina*	*G. vaginalis*	*vaginalis*	96.8 [96.6, 97.1]	81.1 [73.7, 87.5]	82 [76, 87.2]
G. vaginalis	*vaginalis*	*G. vaginalis*	*lacydonensis*	95.9 [95.3, 96.6]	82.1 [74.5, 88.2]	81 [75, 86.2]
G. vaginalis	*vaginalis*	*G. vaginalis*	*regina*	96.9 [96.6, 97.1]	81.1 [73.7, 87.5]	82 [76, 87.2]
G. vaginalis	*vaginalis*	*G. vaginalis*	*vaginalis*	98.4 [98, 99]	91.1 [84.1, 97.2]	92 [87, 97.7]
G. washingtoni		*G. washingtoni*		98.8 [98.8, 98.9]	92.9 [91.8, 94.9]	94 [94, 95.9]

Comment #4

In Fig. 2a,b, I recommend adjusting the minimum ANI value of the heatmap to 94% and 95%. This would make the clustering of species and subspecies more clearly visible in relation to these threshold values and help highlight the distinctions more effectively.

<u>Response to reviewer</u>:

> We thank the reviewer for this suggestion. We agree that adding 94% and 95% ANI divisions in the heatmap better clarifies how species and subspecies cluster relative to these thresholds. As did not add any clarity to the main figure, have included a new Extended Data Figure 3d to more clearly show the 94% - 95% values falling neatly within a species clade which reinforces the lowered threshold for these species.

New Extended Figure 3f:

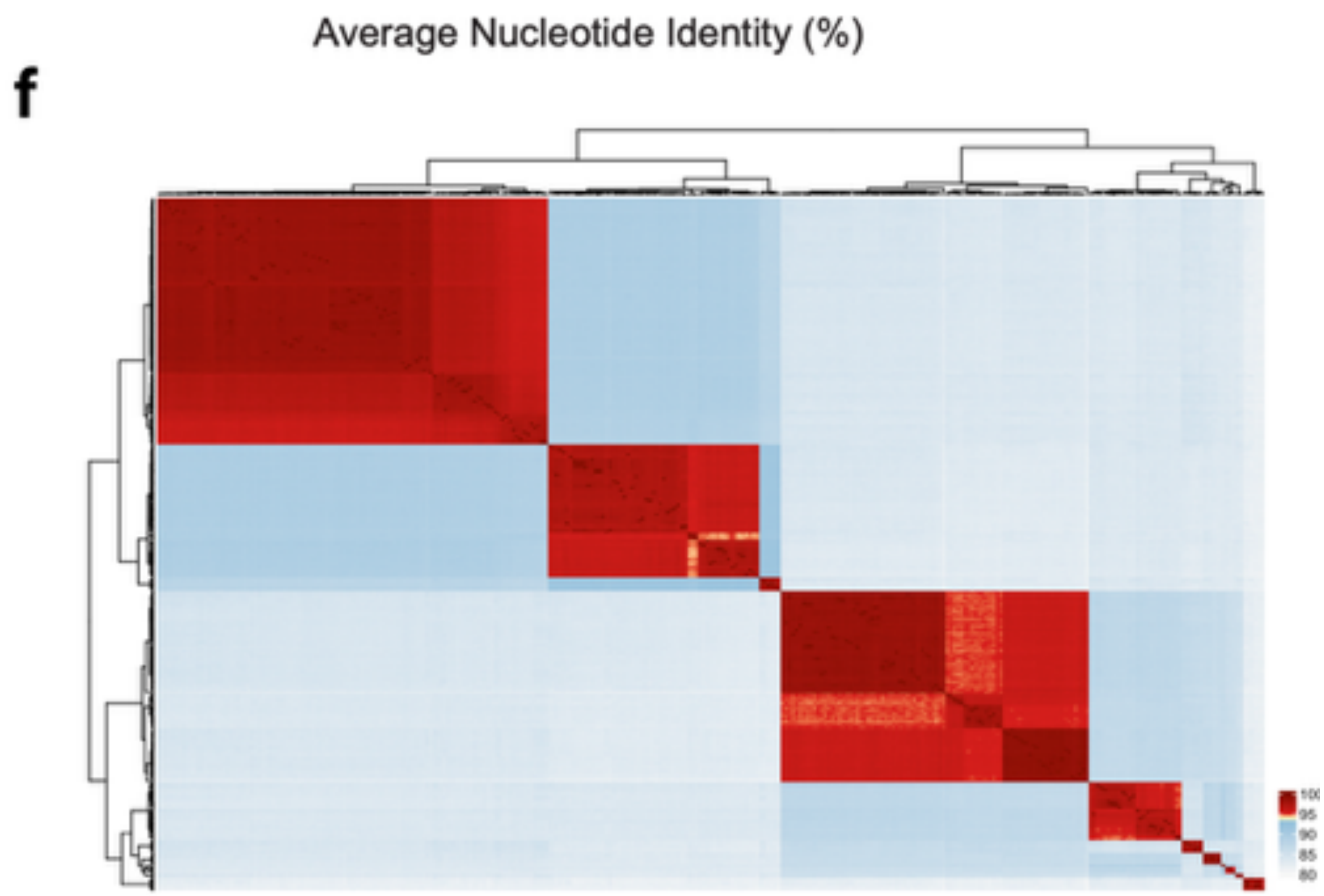

The new caption reads:

Extended Figure 3f, ANI of the *Gardnerella* strains in the curated collection with an emphasis on the division at 95% which is colored white. This emphasizes that the few strains that do fall in the 95-94% range are well within otherwise sharply defined species groups.

Comment #5

The species picketii, piotii, leopoldii, and swidsinskii were previously validated as distinct species through a polyphasic approach and genome-based analyses. It would be helpful if the manuscript addresses how these earlier findings align—or contrast—with the current classification of these taxa as subspecies. Revisiting the ANI and dDDH thresholds in light of the established results could strengthen the argument and ensure consistency with prior taxonomic frameworks. Applying the universally accepted ANI and dDDH thresholds for species delineation would help preserve these established designations and avoid confusion in the naming and classification of Gardnerella.

Response to reviewer:

> We thank the reviewer for highlighting the importance of aligning our classification with previously validated species. We revisited the ANI and dDDH values for *G. pickettii*, *G. piotii*, *G. leopoldii*, and *G. swidsinskii* considering the established thresholds. Our analyses indicate that these taxa form closely related clades within the expanded *Gardnerella* framework, supporting their designation as subspecies under the new classification while remaining consistent with prior polyphasic and genome-based studies. We have updated the manuscript to explicitly discuss this alignment and included the relevant ANI and dDDH values to ensure transparency and consistency with established taxonomic conventions.

The revised work adds this paragraph (Lines 119-129):

The *Gardnerella* species *bretellae, lacydonensis, leopoldii, phocaeensis*, *pickettii, piotii*, and *swidsinskii* were previously described as distinct species when integrating phenotypic characterization with genome-based analyses. In our dataset, these lineages likewise form well-supported monophyletic clades in whole-genome phylogenies and remain clearly differentiated from other *Gardnerella* taxa. However, when evaluated across the expanded genome collection as analyzed here, pairwise ANI and dDDH comparisons reveal that these lineages consistently fall within the genomic coherence of broader species-level clusters defined by ≥95% ANI and ≥70% dDDH thresholds. While they are phylogenetically structured and phenotypically distinguishable, they do not cross the currently accepted genomic discontinuity thresholds that would necessitate recognition as separate species under genome-based standards.

Reviewer 3

Comment #1

The manuscript combines three components that are related; however, the result is somewhat disjointed. The first two components would package well as a taxonomic "renovation" of Gardnerella. The third component describes an exciting discovery and the development of a genetic tool that will be useful to the field.

The title does not mention the novel contribution described in the manuscript – the isolation of a cryptic plasmid and its use in building a shuttle vector for transformation of Gardnerella vaginalis.

Response to reviewer:

Upon consideration of this nuanced comment, we have revised the title of the work to better reflect all major components of the work, including the plasmid and phage discoveries.

The original title was:

'A Syntenic Pangenome for Gardnerella Reveals Taxonomic Boundaries and Stratification of Metabolic and Virulence Potential across Species'

The revised title now reads:

A Syntenic Pangenome of Gardnerella Reveals Novel Plasmids and Phage, Taxonomic Boundaries, and Species-Level Stratification of Metabolic and Virulence Potential

Comment #2

The lack of focus is also evident in the statement of the objective (lines 48-50), which lacks specificity.

Response to reviewer:

We have additionally revised this statement to more explicitly reflect the taxonomic, comparative genomic, and experimental components of the manuscript.

The text originally stated:

'This work aims to advance knowledge on the genomic diversity, genetic potential, species boundaries, and taxonomy within Gardnerella, a genus central to a globally prevalent human condition.'

The revised statement now states (lines 47-50):

Collectively, this study integrates pangenome analyses, genome-based taxonomy, and experimental validation to define species boundaries within *Gardnerella*, characterize lineage-specific metabolic and virulence-associated potential, and identify mobile genetic elements, including a cryptic plasmid that enables the development of a shuttle vector for the genetic engineering of *Gardnerella*.

Comment #3

Changes to the Discussion are needed. The Discussion is largely limited to re-stating the results without relating them to vaginal ecology or BV.

Response to reviewer:

We have now substantially revised the Discussion to better integrate our genomic results with current understanding of vaginal ecology and bacterial vaginosis (BV). Specifically, we now relate lineage-specific metabolic capacities, virulence-associated features, and mobile genetic elements to differences in ecological persistence, community structure, and associations with BV, rather than reiterating the results alone.

The original text stated:

With this refined taxonomic and nomenclature framework, we probed for genetic features and putative operons associated with each lineage by applying a syntenic pangenome approach. Through this and other analyses, we linked lineages to distinct repertoires of virulence factors, metabolic pathways, and defence systems, highlighting functional diversity across the genus. We noted that the vast majority of Gardnerella gene content (~84%) is species and strain-variable with many differences associated with virulence, metabolism, and genetic defence systems. Gardnerella Set A genomes (Gr, Gs, and Gv) showed an enrichment for sialidases and an increased potential for the catabolism of small carbon sources. We further noted lineage-specific enrichment for specific virulence factors (NanH2- Gr and Gspt, NanH3- Gspk and Gspt, NanH4-Gv, vaginolysin-Gvv, Gvt, Gss and Gspk). In contrast, Set B genomes (Ga, Gb, Gc, Gi, Gk, Gm, Gn, and Gw) were enriched in putative adhesion and virulence proteins, displayed a greater capacity for amino acid biosynthesis, had a higher propensity to harbor the vG_gva_AB1 prophage, and contained a broader repertoire of genetic defence mechanisms.

The revised text now states (lines 399-419):

With this refined taxonomic and nomenclature framework, we probed for genetic features and putative operons associated with each lineage by applying a syntenic pangenome approach. These analyses linked *Gardnerella* lineages to distinct repertoires of virulence-associated factors, metabolic pathways, and genetic defense systems, highlighting functional diversity across the genus. Notably, most *Gardnerella* gene content (84%) is species- or strain-variable with many differences related to virulence-associated functions, metabolism, and genetic defense.

Gardnerella Set A genomes (*Gr, Gp,* and *Gv*) were enriched for sialidases and exhibited increased potential for the catabolism of small carbon sources. We further noted lineage-specific enrichment of established virulence-associated factors, including exported sialidases (*nanH2* in *Gr* and *Gppt*; *nanH3* in *Gppk* and *Gppt*) and vaginolysin (enriched in *Gvv, Gvl, Gps* and *Gppk*). The restricted distribution of these factors suggests functional specialization among lineages that may contribute to mucin degradation and epithelial barrier disruption within the vaginal microbiome. In contrast, Set B genomes (*Gb, Gc, Gg, Gi, Gl, Gk, Gn,* and *Gw*) were enriched in putative virulence-associated proteins (e.g. predicted surface-associated proteins, secretion system components and factors implicated in host interaction), exhibited greater capacity for amino acid biosynthesis, showed a higher propensity to harbor the vG_gva_AB1 prophage, and encoded a broader repertoire of genetic defense mechanisms. Collectively, these features are consistent with distinct colonization strategies among Set B *Gardnerella* lineages, including enhanced persistence on epithelial surfaces, flexibility under nutrient-limited or variable resource conditions, and adaptation to competitive, phage-rich, polymicrobial niches.

We added the following paragraph at the end of the Discussion section (lines 433-439):

Although these interpretations are based on comparative genomic potential and do not directly infer *in vivo* activity or clinical outcomes, taken together they support the view of *Gardnerella* as an ecologically diverse genus composed of lineages with distinct metabolic capacities, host-interaction mechanisms, and mobile genetic repertoires. Different *Gardnerella* seem to occupy overlapping but not identical niches within their human hosts. Some lineages may be better adapted to persistence and competitive growth in the vaginal microbiome and urinary tract, whereas others may be more consistent with transient or context-dependent colonization.

Comment #4

***Proposed reclassification of Gardnerella**:*

It is not surprising that as whole genome sequences accumulate, additional subdivisions within existing taxa emerge. It was surprising that given all the effort to define taxonomic boundaries that the authors only went as far as proposing provisional names and did not meet the requirements for valid publication of new taxa as defined in the International Code of Nomenclature of Prokaryotes including deposit of type strains into two culture collections. The authors have put so much work into a carefully curated set of data and a thorough analysis, so it

seems a shame to leave it as "provisional".

Response to reviewer:

We thank the reviewer for this comment and fully agree on the importance of formally validating these taxa.

Hence, we consulted with Dr. Marko Kostovski, a specialist in bacterial nomenclature, to ensure that the proposed names and descriptions are consistent with the requirements of the International Code of Nomenclature of Prokaryotes and current practices of the International Committee on Systematics of Prokaryotes.

We are depositing the relevant type strains in two recognized culture collections (CCUG and DSMZ) and have provided updated protologues required for valid naming. As discussed with Dr. Kostovski, inclusion of these elements in the present manuscript would allow the names to be effectively published, after which a validation request can be submitted to the International Journal of Systematic and Evolutionary Microbiology for inclusion in the Validation Lists and thereby achieve valid naming under the ICNP.

The present manuscript would therefore represent the genomic and phylogenomic phase of a broader proposed taxonomic revision of the genus *Gardnerella*, establishing the genomic framework that supports the proposed reclassification, while the subsequent validation process would formally establish the new taxa. We thank the reviewer for their suggestions and support.

Comment #5

Some of the taxon proposals are not well supported since they are based on <5 isolates. Although it is technically possible to validly publish a new taxon based on a single isolate, multiple isolates (preferably epidemiologically distinct) obviously strengthen the case. Lack of multiple isolates is why Vaneechouette et al. did not propose names for multiple "genome species" identified in their study. A search of available shotgun metagenomic sequence data from vaginal microbiomes might yield at least some in silico support for these taxa and should be considered by the authors.

Response to reviewer:

We thank the reviewer for this suggestion. We agree that inclusion of multiple isolates, ideally from epidemiologically distinct samples, strengthens taxonomic proposals. For this study, we prioritized high-quality cultured genomes to ensure robust genomic characterization and stable reference material.

To address the reviewer's concern regarding limited isolate numbers, we additionally searched public shotgun metagenomic datasets from vaginal microbiomes as well as our in-house metagenome collections. We identified multiple medium-to-high quality MAGs that cluster phylogenomically with the proposed species and subspecies and meet accepted completeness and contamination thresholds. These MAGs provide independent *in silico* support for the genomic coherence of these taxa and partially mitigate the limited number of cultured isolates.

This analysis is now included as Extended Data Figure 3g and described in the Results. While we agree that future recovery of additional cultured isolates will further strengthen these proposals, we believe the combined evidence from cultured genomes and metagenomic data is sufficient to support the taxonomic conclusions presented here.

The new figure appears as Extended Data Figure 6d:

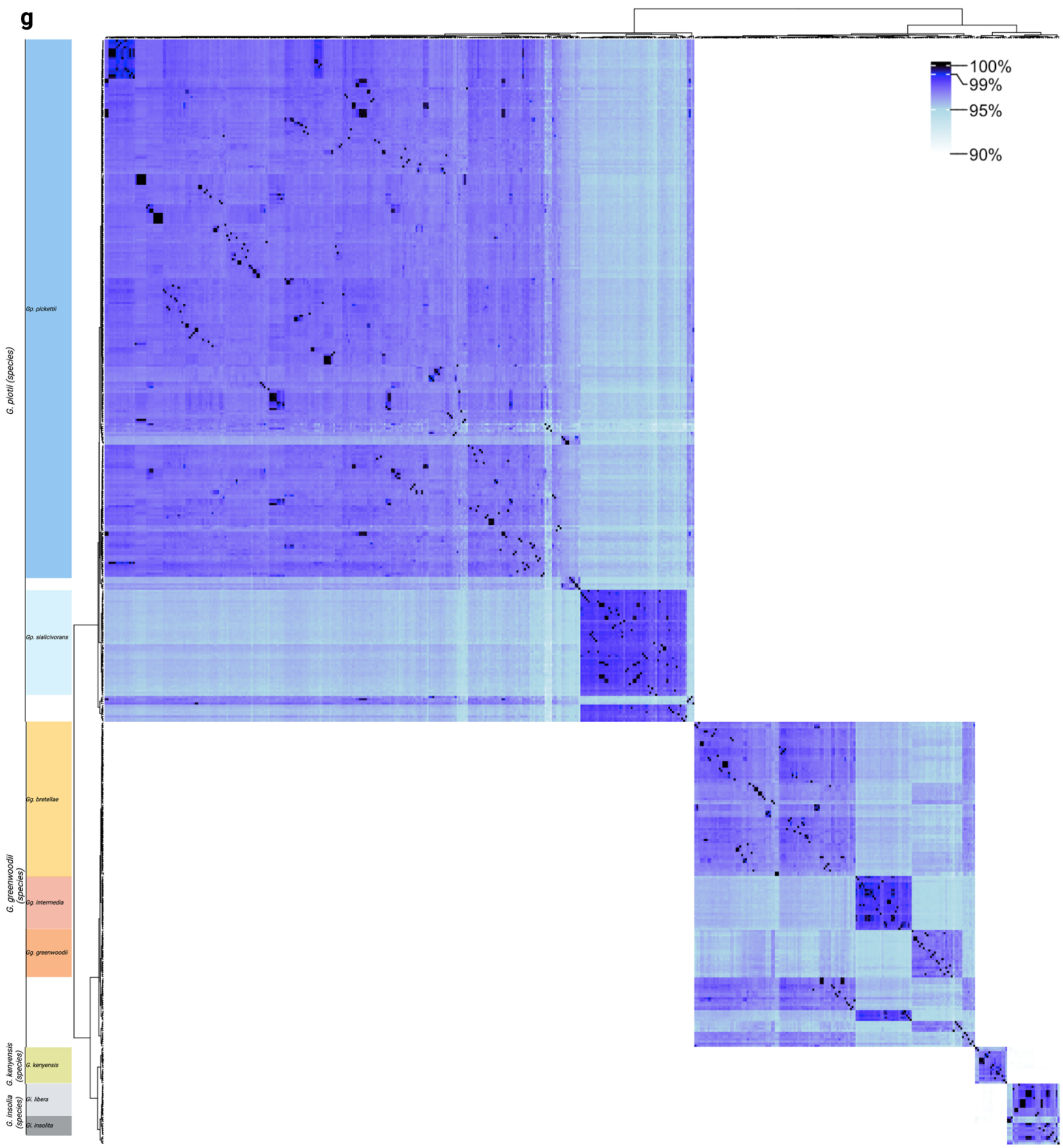

With the new caption:

Extended Figure 3g, Heatmap of pairwise ANI values for species in *Gardnerella* with low numbers of cultured isolates at the subspecies level as compared to MAGs (metagenome assembled genomes). Colored bands at the left give the species and subspecies groups for the boxes in the heatmap. Rows without coloration are to MAGs that are unassigned to species.

Comment #6

Further comment on the Bifidobacterium/Gardnerella distinction is needed – especially as it relates to the GTDB classification and that their framework places Gardnerella within Bifidobacterium despite the obvious and numerous differences (genome size, G+C content, etc.). This is a long-standing issue, the solution for which is most likely to make the case that Bifidobacterium is multiple genera. While beyond the scope of this study, the authors should take the opportunity to comment on/recommend potential resolutions.

Response to reviewer:

> We thank the reviewer for this thoughtful and important comment. We agree that the placement of *Gardnerella* within *Bifidobacterium* (as it is currently within the GTDB) highlights a long-standing and unresolved issue in actinobacterial taxonomy. While GTDB prioritizes genome-scale phylogeny based on concatenated marker genes, this approach can obscure biologically meaningful genus-level distinctions when deeply branching lineages exhibit substantial divergence in genome size, G+C content, gene repertoire, metabolic capacity, and ecological specialization, all of which clearly differentiate *Gardnerella* from canonical *Bifidobacterium* species.
>
> We agree with the reviewer that a likely resolution is recognition that *Bifidobacterium*, as currently circumscribed, encompasses multiple deeply divergent lineages that may warrant separation into multiple genera. However, we also agree that a comprehensive reassessment of genus-level taxonomy across *Bifidobacteriaceae* (which may include expanded sampling of type strains, phenotypic characterization, and more formal nomenclatural actions) is well beyond the scope of the present study.
>
> Accordingly, we retain the genus name *Gardnerella* in this manuscript, consistent with the International Code of Nomenclature of Prokaryotes and current validly published names, while explicitly framing our work as contributing genome-based evidence relevant to future taxonomic revision. We have revised the Discussion to more clearly acknowledge this tension between GTDB-based classification and classical genus concepts, and to highlight the need for a broader, community-driven re-evaluation of genus boundaries within *Bifidobacteriaceae*.

The original discussion stated:

> *With this refined taxonomic and nomenclature framework, we probed for genetic features and putative operons associated with each lineage by applying a syntenic pangenome approach. Through this and other analyses, we linked lineages to distinct repertoires of virulence factors, metabolic pathways, and defence systems, highlighting functional diversity across the genus. We noted that the vast majority of Gardnerella gene content*

(~84%) is species and strain-variable with many differences associated with virulence, metabolism, and genetic defence systems.

The revised discussion now states (lines 383-405):

Our genome-based analyses also intersect with a broader, long-standing debate regarding the placement of *Gardnerella* within the family *Bifidobacteriaceae*. Although, marker gene frameworks such as the GTDB place *Gardnerella* within *Bifidobacterium*, the two groups differ markedly in genome size, G+C content, metabolic repertoire, virulence-associated gene content, mobile genetic element composition, and ecological specialization. Notably, *Gardnerella* genomes are substantially smaller and lack many biosynthetic and metabolic pathways retained in larger *Bifidobacterium* genomes. Such genome reduction is widely interpreted as a hallmark of specialization, reflecting adaptation to host-associated niches where broad metabolic versatility is unnecessary. In this context, reduced genome size represents ecological specialization rather than diminished biological significance.

Together, these genome-scale differences suggest that conserved marker phylogeny alone may be insufficient to define biologically meaningful genus boundaries within this family. A parsimonious interpretation is that *Bifidobacterium*, as currently circumscribed, encompasses multiple genomically and ecologically distinct genera. While a formal genus-level revision of *Bifidobacteriaceae* lies beyond the scope of this study, our data provide additional genome-scale evidence supporting the continued recognition of *Gardnerella* as a distinct genus and contribute to an evidence base that may inform future ICNP-compliant taxonomic restructuring. With this refined taxonomic and nomenclature framework, we probed for genetic features and putative operons associated with each lineage by applying a syntenic pangenome approach. These analyses linked *Gardnerella* lineages to distinct repertoires of virulence-associated factors, metabolic pathways, and genetic defense systems, highlighting functional diversity across the genus. Notably, most *Gardnerella* gene content (84%) is species- or strain-variable with many differences related to virulence-associated functions, metabolism, and genetic defense.

Comment #7

The effort of the authors to provide historical perspective on the taxonomy of Gardnerella is appreciated, however, there is some clarification needed. In the section starting on line 80:

- (line 90) The citation for the subgroup A-D system is incorrect and this system was not based on whole genome sequencing. This system was based on cpn60 barcode sequencing and introduced by Jayaprakash et al. (2012) (ref 36).

- (line 92) "Nearly simultaneously…" clades 1-4 were described by Ahmed et al. (2012) (ref 30, not 42). The reference to Balashov et al. 2014 is also not relevant here.

- (line 101) refers to prior studies that use the "Set A and Set B" terminology but only one study is cited (30). If there are other examples of this usage (not sure there are), they should be included

- Figure 1A is helpful but Figure 1B is confusing as presented. What is the basis for the lines connecting Piot/Benito biotypes with the cpn60 subgroup/WGS clade terms? Both Jayaprakash

et al. and Ahmed et al. reported Piot biotypes in their studies but those results do not align with Figure 1B
- Figure 1B – in the cpn60 subgroup/WGS clade column what are "B3", "A2", etc. (no slashes?)

Response to reviewer:

We thank the reviewer for their careful analysis of this section and for identifying several inaccuracies and points requiring clarification. Historical precision is essential when summarizing prior taxonomic frameworks, and we have revised both the text and Figure 1 accordingly.

1. **Subgroup A–D system (line 91)**

   The citation and description were incorrect. The A–D subgroup system was introduced by Jayaprakash *et al*. (2012) and was based on cpn60 barcode sequencing, not whole-genome sequencing. We have corrected the citation to reference Jayaprakash *et al*. and revised the text to accurately describe the methodological basis of this classification.
   The text on lines 89-91 now reads:

   PCR and whole-genome sequence comparisons introduced two new naming schemes including designations "A - D"[12] or "1 - 4"[2] which led to hybrid labels ("C/1", "B/2", "A/4", and "D/3")[13].

2. **Clades 1–4 (line 92)**

   The wording and citations were inaccurate. The clades 1–4 system was described by Ahmed et al. (2012), not Balashov et al. (2014). We have corrected the reference and revised the phrasing to accurately reflect the historical sequence and source of this classification.

3. **Use of "Set A" and "Set B" terminology (line 101)**

   We agree that the terminology "Set A" and "Set B" has not been broadly adopted in prior studies. We have revised the text to clarify that these labels were introduced by Ahmed *et al*. and that our intent is to highlight a consistently recovered basal bifurcation in *Gardnerella* phylogenies, rather than to imply formal recognition of these groups as named clades across the literature.

   The text on lines 97-101 now reads:

Despite the use of diverse classification schemes, phylogenetic analyses across studies consistently recover a deep bifurcation within *Gardnerella*, with the same two major lineages forming the basal split of the genus. While this division has been explicitly designated as "Set A" and "Set B" only

by Ahmed *et al*., analogous groupings confirm the primary branching structure in multiple independent phylogenetic frameworks[2,4-7,12,14,18-20].

4. **Figure 1B: connections between Piot/Benito biotypes and later classifications**
   We agree that Figure 1B, as originally presented, inadvertently implied a level of concordance between Piot/Benito biotypes and later cpn60 or genome-based groupings that is not fully supported by the literature. Jayaprakash *et al*. and Ahmed *et al*. reported Piot biotypes, but these did not map cleanly onto later subgroup or clade assignments. We have revised Figure 1B to clarify that these connections are illustrative rather than deterministic, and we have updated the figure legend to explicitly state the limitations and lack of strict correspondence among these classification systems.

   The original caption read:
   Lineages consistent between different publications are connected by arrows.

   The revised caption now reads:
   Arrows indicate consistent lineages between studies without implying a definitive link.

5. **Figure 1B: meaning of "B3", "A2", etc.**
   We agree that these labels were insufficiently defined. These designations refer to the manner in which Ahmed *et al*. discussed these groups. These secondary labels have been removed and instead the secondary designation is only noted in the caption to the figure.

The revised Figure 1 now appears like this:

REDACTED

Comment #8

The authors report using a 94% ANI threshold for designating "species" but a description of the threshold/requirements for defining "subspecies" should be provided. Also, please explain the rationale for using 94%.

Response to reviewer:

We thank the reviewer for raising this important point and note that Reviewer #2 also commented on this threshold choice, highlighting the need to address it explicitly. The 95% ANI threshold commonly used for species delineation is based on early large-scale genome comparisons demonstrating a pronounced discontinuity in pairwise ANI values, with most interspecies comparisons falling below ~83% and intraspecies comparisons clustering above ~95%. This empirical "ANI gap" was originally noted to exclude approximately 5–6% of genome comparisons and has since been widely adopted as a practical operational definition for bacterial species (https://doi.org/10.1038/s41467-018-07641-9).

Subsequent studies have documented biologically meaningful cases in which closely related taxa consistently occupy the upper edge of this gap, with stable and phylogenetically coherent lineages exhibiting ANI values slightly below 95%. Such cases

are particularly evident among recently diverged, phage-associated, or host-adapted bacteria(https://doi.org/10.1099/ijsem.0.004124; https://doi.org/10.1073/pnas.0409727102; https://doi.org/10.1111/jeu.12944).

In our curated *Gardnerella* dataset, multiple genomically cohesive and phylogenetically supported lineages consistently clustered above 94% ANI and were concordant with phylogeny, gene content, and synteny. Only the smallest fraction of the sequences fell below 95% as could be observed in Extended Figure 3a. As removing these sequences based on a threshold that was not founded in biological observation seemed arbitrary, we adopted a conservative 94% ANI threshold to delineate species-level boundaries while minimizing the subdivision of closely related taxa.

Subspecies designations were assigned to lineages that formed well-supported monophyletic groups within species-level clusters (ANI ≥94%) but remained distinguishable by consistent differences in genome content, syntenic organization, and digital DNA–DNA hybridization (dDDH) values. dDDH comparisons were used to confirm congruence between ANI-based clustering and subspecies-level divergence.

We have revised the manuscript to explicitly describe these criteria and to clarify the rationale for both species- and subspecies-level thresholds.

The original text read:

To determine the number of Gardnerella species represented, we compared the genome-wide ANI values and identified a 94% threshold that delineated intra-species comparisons (Extended Data Fig. 3a), slightly below the prokaryotic species ANI threshold of 95 ± 0.5 % reported by Jain et al.

The revised text now reads (lines 107-115):

In accordance with prior large-scale analyses demonstrating a pronounced discontinuity in ANI values around the canonical prokaryotic boundary, most inter-lineage comparisons fell well below 95%, whereas closely related genomes clustered above 95% with an exceedingly small fraction of the sequence comparisons falling above 94% (Extended Data Fig. 3a). Because ANI and digital DNA–DNA hybridization (dDDH) values were strongly colinear across the dataset, dDDH did not provide additional resolution but independently supported the same groupings: all genomes that clustered together by ANI also exceeded the 70% dDDH species threshold (Extended Data Fig. 3b–c). Thus, species boundaries inferred from ANI were fully congruent with the universally accepted 70% dDDH.

Comment #9

Lines 123-131. The suggested shorthand for all the proposed species and subspecies is not consistent with rules of nomenclature and should be removed. Use of the shorthand within the

manuscript is acceptable if explained.

Response to reviewer:

We appreciate this helpful comment. We agree that the shorthand using in the main text should not be extended to the protologues. Any references to the abbreviations was removed from the Protologues and better explained in the text.

The original text read:

> *Species names are to be abbreviated with two letters (Ga, Gb, Gc, Gi, Gk, Gm, Gn, Gr, Gs, Gv and Gw), while individual subspecies should be designated with three-letter abbreviations (except for Gs subsp. pickettii and Gs subsp. piotii, which require four letters, Gspk and Gspt, to distinguish them from other named subspecies) (Fig. 1, Supplementary Table 4, Methods).*

The revised text now reads (lines 141-146):

To minimize ambiguity in referring to *Gardnerella* species in this paper and future efforts, we intentionally propose species names that can be abbreviated with unique two letter abbreviations (*Gb*, *Gg, Gh, Gi*, *Gk*, *Gl*, *Gm, Gp, Gr*, *Gv* and *Gw*), while individual subspecies were designated with discernable three-letter abbreviations (except for *Gardnerella piotii* subsp. *pickettii* and *Gardnerella piotii* subsp. *piotii,* which each require four letters, *Gppk* and *Gppt*) (Fig. 1, Supplementary Table 4).

Comment #10

Pangenome analysis: *The authors present a report of genes encoding virulence factors and predicted metabolic capabilities across the Gardnerella lineages. The results confirm current understanding that there are not strictly "pathogenic" and "non-pathogenic" Gardnerella, that there is diversity in metabolic features and the distribution of defence mechanisms like restriction modification systems. The results presented are generated in silico with no phenotypic evidence to support the conclusions and no specific hypotheses are presented or tested. The Discussion does not relate these findings to ecology in the vaginal microbiome or to BV and current models of etiology.*

Response to reviewer:

> The goal of this section was to integrate functional genomic features within the revised genome-resolved taxonomy and nomenclature established in this study.

Presenting an updated taxonomy/nomenclature alone would leave much of the existing biological literature disconnected from the revised lineage definitions. We therefore consider it essential to map established predicted virulence-associated genes, metabolic pathways, and defence systems across the resolved lineages using a syntenic pangenome framework. While individual genes and pathways have been reported previously, their distribution has not been examined within this unified and internally consistent taxonomic structure.

Linking these genomic features to the revised nomenclature provides the biological context necessary for interpreting lineage-level differences and establishes a framework that future studies can use to investigate *Gardnerella* ecology and its role in bacterial vaginosis.

Additionally, as described in our response to Reviewer 2, Comment #3, the Discussion has been substantially revised to relate the genomic findings to current models of vaginal ecology and bacterial vaginosis. In particular, we now interpret lineage-specific differences in metabolic capacity, virulence-associated factors, and mobile genetic elements in terms of ecological strategies within the vaginal microbiome (e.g., mucin degradation, epithelial interaction, persistence under nutrient limitation, and adaptation to polymicrobial and phage-rich environments).

<u>Comment #11</u>

Line 201-204 – Reference 51 does not mention NanH4. NanH4 was described by Pelayo et al. PNAS (2024) as being encoded by a gene found in a minority of isolates and importantly it had no signal peptide and no association with sialidase activity. Similarly, NanH1 ("Sialidase A") is not associated with sialidase activity and is likely intracellular. Unfortunately, PCR for NanH1 was used for several years as evidence of "sialidase positive" Gardnerella isolates. If the point is to look at distribution of virulence factors, this analysis should be limited to NanH2 and NanH3.

*Along this same avenue, the observation that Set A genomes showed an "enrichment for sialidases" (line 380) is somewhat misleading since sialidase *activity* is largely restricted to Gs piotii and absent from G. vaginalis. This reflects the distribution of NanH2 and NanH3.*

<u>Response to reviewer</u>:

We thank the reviewer for this important clarification regarding NanH family annotations and functional activity.

In response, we have revised the manuscript to (i) correct the citation regarding NanH4, (ii) clarify that NanH1 and NanH4 are not associated with extracellular sialidase activity,

and (iii) restrict our functional interpretation of sialidase-associated virulence to NanH2 and NanH3, which account for the majority of extracellular sialidase activity in *Gardnerella*. References to "sialidase genes" have been modified where appropriate to explicitly distinguish between gene presence and demonstrated enzymatic activity.

Regarding the statement that Set A genomes showed an "enrichment for sialidases," we agree that this phrasing could be misleading, as extracellular sialidase activity is largely restricted to *G. piotii* subspecies *piotii* and is generally absent from *G. vaginalis* subspecies *vaginalis*, reflecting the distribution of *nanH2* and *nanH3*. We have therefore revised this section to specify that the enrichment pertains to the genomic presence of *nanH2*/*nanH3* homologs rather than inferred enzymatic activity, and we now explicitly note the lineage-restricted distribution of extracellular sialidase activity.

These revisions clarify both the functional distinctions among *nanH* paralogs and the lineage-specific nature of extracellular sialidase activity in *Gardnerella*.

The previous text read:

Canonical Gardnerella virulence factors include four sialidase variants (NanH1, NanH2, NanH3, NanH4) aiding in mucin degradation when exported, and vaginolysin, which binds to and lyses host cells in the vaginal mucosa In our pangenome dataset we identified specific nodes associated with each of these virulence factors (Fig. 4d-e, Extended Data Fig. 5, Supplementary Table 5).

(and)

We further noted lineage-specific enrichment for specific virulence factors (nanH2- Gr and Gspt, nanH3- Gspk and Gspt, nanH4-Gv, vaginolysin-Gvv, Gvt, Gss and Gspk).

The current text now reads (lines 211-220):

Among *Gardnerella*-associated sialidase isoforms, NanH2 and NanH3 account for the majority of extracellular sialidase activity, whereas NanH1 lacks a signal peptide and is therefore predicted to function intracellularly for sugar catabolism[21]. Consistent with this role, *nanH1*[22] is embedded within a conserved operon across genomes (Extended Figure 5e). In contrast, *nanH2* and *nanH4*[23] are frequently located in proximity to this operon but are associated with mobile genetic elements, suggesting more recent paralogous diversification and horizontal acquisition. Notably, *nanH3* is uniquely not operon-associated; instead, it is found as a solitary gene within defense-associated genomic regions, often adjacent to prophage elements, consistent with a distinct evolutionary trajectory and potential phage-linked mobility.

(and on lines 407-419)

We further noted lineage-specific enrichment of established virulence-associated factors, including exported sialidases (*nanH2* in *Gr* and *Gppt*; *nanH3* in *Gppk* and *Gppt*) and vaginolysin (enriched in *Gvv, Gvl, Gps* and *Gppk*). The restricted distribution of these factors suggests functional specialization among lineages that may contribute to mucin degradation and epithelial barrier disruption within the vaginal microbiome. In contrast, Set B genomes (*Gb, Gc, Gg, Gi, Gl, Gk, Gn,* and *Gw*) were enriched in putative virulence-associated proteins (e.g. predicted surface-associated proteins, secretion system components and factors implicated in host interaction), exhibited greater capacity for amino acid biosynthesis, showed a higher propensity to harbor the vG_gva_AB1 prophage, and encoded a broader repertoire of genetic defense mechanisms. Collectively, these features are consistent with distinct colonization strategies among Set B *Gardnerella* lineages, including enhanced persistence on epithelial surfaces, flexibility under nutrient-limited or variable resource conditions, and adaptation to competitive, phage-rich, polymicrobial niches.

Comment #12

Lines 207-208. "type I pullulanase" should be replaced with "type II α-amylase-pullulanase" as described in the reference cited (54)

Response to reviewer:

We thank the reviewer for this correction. The text in the caption in line 203 has been revised to replace "type I pullulanase" with "type II α-amylase–pullulanase," in accordance with the cited reference.

(and on line 226-227):

A Type II α-amylase-pullulanase protein was well conserved (present in 100% of *Gardnerella* genomes), in agreement with previous reports[24] (Fig. 4d).

Comment #13

The Set B genomes are described on line 384 as enriched for "putative adhesion and virulence proteins". Please clarify as there is not mention of adhesion elsewhere and "virulence proteins" is vague.

Response to reviewer:

We have revised the text to explicitly define what is meant by "putative adhesion and virulence proteins," specifying the functional annotations and protein families underlying

this designation (e.g., predicted surface-associated proteins, secretion system components, and factors implicated in host interaction). In addition, we have clarified the basis for these annotations in the Results and Methods sections and removed overly broad terminology where more precise descriptions can be made.

The original text read:

In contrast, Set B genomes (Gb, Gc, Gg, Gi, Gl, Gk, Gn, and Gw) were enriched in putative adhesion and virulence proteins

The current text on lines 412-414 now reads:

In contrast, Set B genomes (*Gb, Gc, Gg, Gi, Gl, Gk, Gm,* and *Gw*) were enriched in putative virulence-associated proteins (e.g. predicted surface-associated proteins, secretion system components and factors implicated in host interaction)

Comment #14

Cryptic plasmid *This is most exciting and novel aspect of the manuscript although the results are under-described and the methods are not sufficiently complete to allow readers to reproduce the work. For example, the actual conditions used in the "optimized" transformation are not specifically described. Publication of a laboratory protocol in an open access repository would be a service to the field.*

Response to reviewer:

We agree that the identification of the cryptic plasmid and its application for genetic manipulation of *Gardnerella* represent a particularly novel and valuable contribution, and we appreciate the reviewer's emphasis on reproducibility. We have revised both the Results and Methods sections to provide a substantially expanded description of the plasmid, vector construction, and transformation workflow. This includes explicit reporting of the transformation conditions used in the optimized protocol (cell growth phase, DNA amounts, buffer composition, electroporation parameters, recovery conditions, and selection criteria).

In addition, to facilitate reproducibility and broader adoption by the community, we have prepared a detailed laboratory protocol describing the transformation procedure and plasmid handling steps and have deposited this protocol in an open-access repository https://www.protocols.io/private/1A47BAD2F1AC11F09AA30A58A9FEAC02. The accession link is now provided in the Methods section. We have also revised the manuscript text to clearly distinguish between exploratory optimization steps and the finalized, reproducible protocol.

Finally, we have revised the manuscript title to explicitly highlight the discovery of the cryptic plasmid and its utility as a genetic tool, improving the visibility and accessibility of this contribution.

The original text in the Results section read:

ATCC 14018 (Extended Data Fig. 9d). While this yielded no transformants, we predicted that overcoming RM defences present in these strains would increase transformation efficiency. A previously reported RM system modifying "GGCC" sites has been described in ATCC 14018[84],and bypassing this system was shown to improve transformation efficiency with suicide-based vectors[79]. Thus, we used commercially available HaeIII methyltransferase (MTase) to methyl-modify these sites in pG01-MC prior to transformation. In combination with further optimization of growth and transformation conditions, transformation efficiency was substantially increased through these approaches from a minimum of $2.30x10^{0}$ transformants per µg of DNA to $6.85x10^{2}$ (Extended Data Fig. 9d).

The revised text on lines 352-361 now reads:

The strain ATCC 14018^{T} encodes a restriction-modification (RM) system which modifies the "GGCC" motif[84] and has been shown to limit transformation efficiency[25]. Thus, we used commercially available *HaeIII* methyltransferase (MTase) to methyl-modify these sites in pG01-MC prior to transformation. Electroporation with methylated pG01-MC successfully transformed *Gvv* ATCC 14018^{T}, but at a very low efficiency (two transformants per µg of DNA). Further optimization of the protocol increased efficiency to 685 transformants per µg of DNA by transforming with 0.5 µg total *HaeIII*-treated vector on competent cells grown with 100 mM NaCl to an OD_{600} of 0.32 and washed with 10% glycerol (Extended Data Fig. 9d). A detailed, step-by-step version of the optimized protocol is available online at (https://www.protocols.io/private/1A47BAD2F1AC11F09AA30A58A9FEAC02).

Comment #15

The sequences of the plasmids shown in Figure 6D must be provided.

Response to reviewer:

We thank the reviewer for this suggestion. The full sequences of the plasmids shown in Fig. 6d are provided in the Supplementary Table 10 and are referenced in the updated figure legend.

The original text read:

[6]d, Schematics depict plasmid annotations for pDNF1199S-01, 297 p0756_546_1_1_BHK10-01, and pNR047-01.

The current text on lines 309-311 now reads:
[6]d, Schematics depict plasmid annotations for pDNF01199S-01, p0756_546_1_1_BHK10-01, and pNR047-01. Full sequences for the plasmids are available in Supplementary Table 10.

Comment #16

The diagram of pDNF01199S-01 in Figure 6D does not match what is shown in extended data Figure 9 where a helicase and toxin-antitoxin system are annotated. This should be clarified in the text and figures.

Response

We thank the reviewer for noting this inconsistency. The original annotations in Extended Data Fig. 9 were based on preliminary nucleotide-level similarity searches that suggested possible helicase and toxin-antitoxin functions. Upon re-evaluation using more stringent protein-level similarity searches (BLASTp), these assignments were not supported with sufficient confidence.

We therefore revised the annotations to reflect the current evidence and now designate these open reading frames as hypothetical proteins. The supplementary figures and annotations have been updated accordingly so that they are consistent with Fig. 6D. At present, there is insufficient evidence to confidently assign helicase or toxin–antitoxin functions to these genes.

The original figure for plasmids in Figure 6d appeared as:

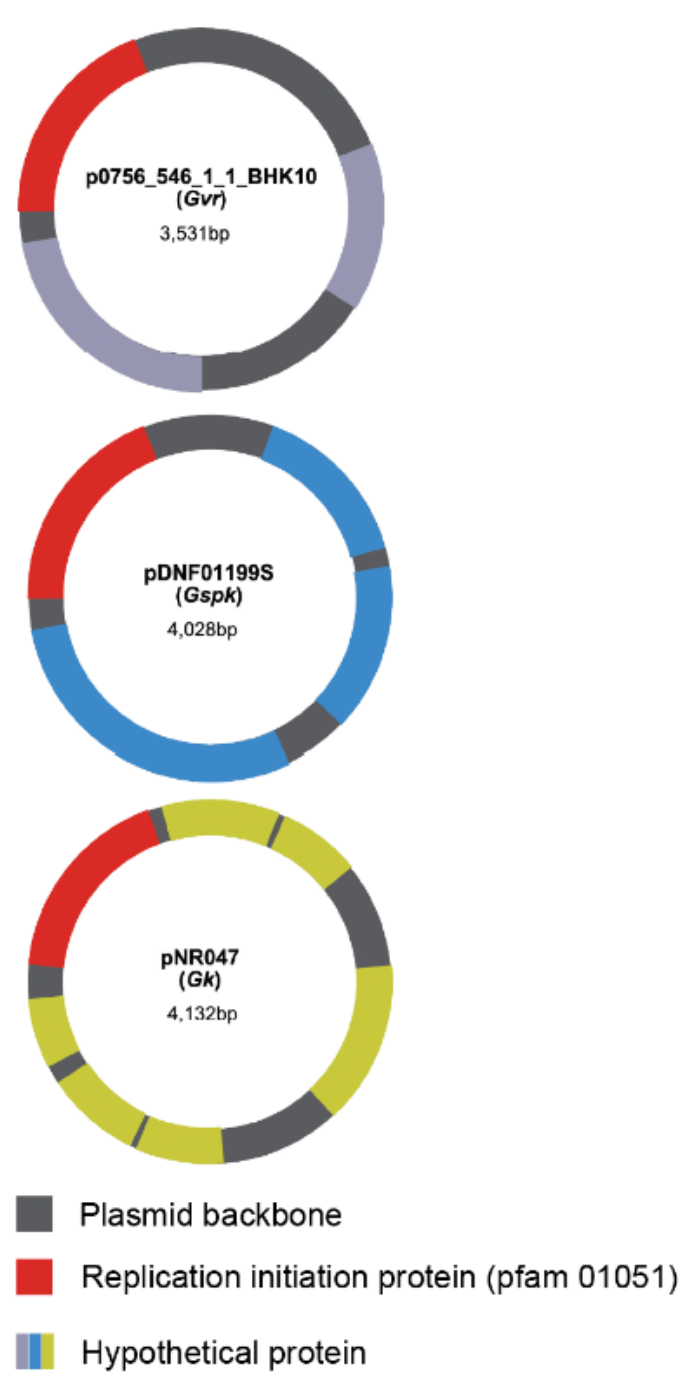

The current Figure 6d now appears as:

REDACTED

Extended Figure 9 previously appeared as:

REDACTED

Extended Figure 9 now appears as:

REDACTED

Supplementary Table 11 shows the result of the BLAST searches for the genes on these plasmids.

Minor Comments

1. *The term "standardized" is used in several places with no definition, e.g. line 149 "standardized names". Suggest removing all of these modifiers*

   The term "standardized" was removed and replaced with other neutral terms.

   The original text read: "*Given the extensive history of conflicting classification schemes (Fig. 1), we next sought to standardize species and subspecies names across all lineages identified*"

   The revised text now on lines 130-133 reads:

Given the extensive history of overlapping and partially incompatible classification schemes for *Gardnerella* (Fig. 1), we next harmonized existing species and subspecies names and established a new nomenclature across all identified lineages (Fig. 1, Fig. 2, Supplementary Tables 2 and 4).

The original text read: "*After stringently identifying the Gardnerella species and subspecies lineages in our genome set and assigning each a standardized name*"

The altered text on lines 161-163 now reads:

Having stringently identified *Gardnerella* species and subspecies lineages in our genome set and assigning names that adhered as closely to precedent, where possible, we were next able to investigate the genetic features associated with each lineage.

2. *The term "unified" is used in the opening paragraph of the Discussion (unified taxonomic framework, unified Gardnerella nomenclature) without definition. Suggest removing these modifiers.*

   The term "unified" was removed with other terms:

   The original text read:

   Using a final curated set of 312 distinct and high-quality genomes, we defined clear lineage boundaries and provide a unified taxonomic framework and propose an updated and unified Gardnerella nomenclature that is reflective of these lineages.

   The new text on lines 370-372 now reads:

Using a curated set of 313 distinct, high-quality genomes, we define clear lineage boundaries and propose an updated *Gardnerella* nomenclature that reflects genome-scale divergence while preserving continuity with existing naming conventions.

3. *Software and database version numbers should be provided throughout.*

   Software version numbers or access dates are now added after the first mention of that software in the Methods section.

1 Janulaitiene, M. *et al.* Prevalence and distribution of Gardnerella vaginalis subgroups in women with and without bacterial vaginosis. *BMC Infectious Diseases* **17** (2017). https://doi.org/10.1186/s12879-017-2501-y

2 Ahmed, A. *et al*. Comparative Genomic Analyses of 17 Clinical Isolates of Gardnerella vaginalis Provide Evidence of Multiple Genetically Isolated Clades Consistent with Subspeciation into Genovars. *Journal of Bacteriology* **194**, 3922-3937 (2012). https://doi.org/10.1128/jb.00056-12

3 Schellenberg, J. J. *et al*. Gardnerella vaginalis Subgroups Defined by cpn60 Sequencing and Sialidase Activity in Isolates from Canada, Belgium and Kenya. *PLOS ONE* **11**, e0146510 (2016). https://doi.org/10.1371/journal.pone.0146510

4 Vaneechoutte, M. *et al*. Emended description of Gardnerella vaginalis and description of Gardnerella leopoldii sp. nov., Gardnerella piotii sp. nov. and Gardnerella swidsinskii sp. nov., with delineation of 13 genomic species within the genus Gardnerella. *Int J Syst Evol Microbiol* **69**, 679-687 (2019). https://doi.org/10.1099/ijsem.0.003200

5 Cornejo, O. E., Hickey, R. J., Suzuki, H. & Forney, L. J. Focusing the diversity of <i>Gardnerella vaginalis</i> through the lens of ecotypes. *Evolutionary Applications* **11**, 312-324 (2018). https://doi.org/10.1111/eva.12555

6 Potter, R. F., Burnham, C. D. & Dantas, G. In Silico Analysis of Gardnerella Genomospecies Detected in the Setting of Bacterial Vaginosis. *Clin Chem* **65**, 1375-1387 (2019). https://doi.org/10.1373/clinchem.2019.305474

7 Bohr, L. L., Mortimer, T. D. & Pepperell, C. S. Lateral Gene Transfer Shapes Diversity of Gardnerella spp. *Front Cell Infect Microbiol* **10**, 293 (2020). https://doi.org/10.3389/fcimb.2020.00293

8 Piot, P. *et al*. Biotypes of Gardnerella vaginalis. *Journal of Clinical Microbiology* **20**, 677-679 (1984). https://doi.org/10.1128/jcm.20.4.677-679.1984

9 Benito, R., Vazquez, J. A., Berron, S., Fenoll, A. & Saez-Nieto, J. A. A Modified Scheme for Biotyping Gardnerella Vaginalis. *Journal of Medical Microbiology* **21**, 357-359 (1986). https://doi.org/https://doi.org/10.1099/00222615-21-4-357

10 Nath, K., Devlin, D. & Beddoe, A. M. Heterogeneity in restriction patterns of Gardnerella vaginalis isolates from individuals with bacterial vaginosis. *Research in Microbiology* **143**, 199-209 (1992). https://doi.org/https://doi.org/10.1016/0923-2508(92)90009-D

11 Ingianni, A., Petruzzelli, S., Morandotti, G. & Pompei, R. Genotypic differentiation of<i>Gardnerella vaginalis</i>by amplified ribosomal DNA restriction analysis (ARDRA). *FEMS Immunology & Medical Microbiology* **18**, 61-66 (1997). https://doi.org/10.1111/j.1574-695x.1997.tb01028.x

12 Jayaprakash, T. P., Schellenberg, J. J. & Hill, J. E. Resolution and Characterization of Distinct cpn60-Based Subgroups of Gardnerella vaginalis in the Vaginal Microbiota. *PLoS ONE* **7**, e43009 (2012). https://doi.org/10.1371/journal.pone.0043009

13 Hill, J. E. & Albert, A. Y. K. Resolution and Cooccurrence Patterns of Gardnerella leopoldii, G. swidsinskii, G. piotii, and G. vaginalis within the Vaginal Microbiome. *Infection and Immunity* **87** (2019). https://doi.org/10.1128/iai.00532-19

14 Tarracchini, C. *et al*. Assessing the Genomic Variability of Gardnerella vaginalis through Comparative Genomic Analyses: Evolutionary and Ecological Implications. *Applied and Environmental Microbiology* **87** (2020). https://doi.org/10.1128/aem.02188-20

15 Gardner, H. L. & Dukes, C. D. Haemophilus vaginalis vaginitis: a newly defined specific infection previously classified non-specific vaginitis. *Am J Obstet Gynecol* **69**, 962-976 (1955).

16 Sousa, M. *et al*. Gardnerella pickettii sp. nov. (formerly Gardnerella genomic species 3) and Gardnerella greenwoodii sp. nov. (formerly Gardnerella genomic species 8) isolated from female urinary microbiome. *Int J Syst Evol Microbiol* **73** (2023). https://doi.org/10.1099/ijsem.0.006140

17 Allini Ntiguemassa, P. A. *et al*. Further dissection of Gardnerella vaginalis: description of Gardnerella lacydonensis sp. nov. (formerly genomic species 2), Gardnerella bretellae sp. nov. (formerly genomic species 9), Gardnerella massiliensis sp. nov. (formerly genomic species 14) and Gardnerella phocaeensis sp. nov. *International Journal of Systematic and Evolutionary Microbiology* **76** (2026). https://doi.org/https://doi.org/10.1099/ijsem.0.007028

18 Innamorati, K. A. *et al*. Metronidazole response profiles of Gardnerella species are congruent with phylogenetic and comparative genomic analyses. *Genome Medicine* **17** (2025). https://doi.org/10.1186/s13073-025-01446-4

19 Teixeira, P., Sousa, M., Ribeiro, T. G., Grosso, F. & Peixe, L. *Unraveling the<i>Gardnerella</i>Pangenome: Insights into Genetic Diversity, Virulence, and Antibiotic Resistance* (Cold Spring Harbor Laboratory, 2025).

20 Malki, K. *et al*. Genomes of Gardnerella Strains Reveal an Abundance of Prophages within the Bladder Microbiome. *PLOS ONE* **11**, e0166757 (2016). https://doi.org/10.1371/journal.pone.0166757

21 Robinson, L. S., Schwebke, J., Lewis, W. G. & Lewis, A. L. Identification and characterization of NanH2 and NanH3, enzymes responsible for sialidase activity in the vaginal bacterium Gardnerella vaginalis. *Journal of Biological Chemistry* **294**, 5230-5245 (2019). https://doi.org/10.1074/jbc.ra118.006221

22 Agarwal, K. & Lewis, A. L. Vaginal sialoglycan foraging by<i>Gardnerella vaginalis</i>: mucus barriers as a meal for unwelcome guests? *Glycobiology* **31**, 667-680 (2021). https://doi.org/10.1093/glycob/cwab024

23 Pelayo, P. *et al*. <i>Prevotella</i> are major contributors of sialidases in the human vaginal microbiome. *Proceedings of the National Academy of Sciences* **121** (2024). https://doi.org/10.1073/pnas.2400341121

24 Bhandari, P., Tingley, J., Abbott, D. W. & Hill, J. E. Glycogen-Degrading Activities of Catalytic Domains of α-Amylase and α-Amylase-Pullulanase Enzymes Conserved in <i>Gardnerella</i> spp. from the Vaginal Microbiome. *Journal of Bacteriology* **205** (2023). https://doi.org/10.1128/jb.00393-22

25 Klimowicz, A. K., Garcia, E. M., Jefferson, K. K. & Dillard, J. P. Genetic transformation of <i>Gardnerella</i> species and characterization of vaginolysin and sialidase mutants. *Infection and Immunity* **93** (2025). https://doi.org/10.1128/iai.00299-25

We thank the editorial team and reviewers for their careful evaluation of our manuscript resubmission. We appreciate the additional constructive comments and have further revised the manuscript to address each new point raised. Below, we respond to each comment in detail, with changes indicated by page and line numbers in the revised manuscript.

Reviewer 1

Comment #1

The revised manuscript is improved and a very comprehensive and useful study.

Response :

> We thank the reviewer for their positive assessment of the revised manuscript. We appreciate the recognition that the study is now improved, comprehensive, and useful. We appreciate the reviewer's suggestions which helped strengthen the clarity and overall quality of the manuscript.

Reviewer 2

Comment #1

The authors have substantially addressed my previous concerns and incorporated my suggestions. The responses to my review comments were comprehensive and satisfactory. The manuscript has undergone significant improvement in both clarity and rigor, and the revisions have strengthened the overall impact of the work. I have no further concerns.

Response :

> We sincerely thank the reviewer for their thoughtful evaluation of the revised manuscript and for acknowledging the improvements made in response to the previous review comments. We appreciate the reviewer's recognition that the revisions strengthened the overall impact of the work. The reviewer's constructive feedback was invaluable in improving the quality of the manuscript, and we are grateful for their careful review and support.

Reviewer 3

Comment #1

Thanks to the authors for carefully considering and responding to the reviewer comments. The manuscript is much improved. I have only a couple of minor comments.

*In response to my comment #3, please replace "infer" with "imply" in the revised text where it says "Although these interpretations are based on comparative genomic potential and do not directly *infer* in vivo activity or clinical outcomes..."*

Response :

The use of “infer” in the specified line has now been replaced with “imply”.

The original text (lines 433-436):

Although these interpretations are based on comparative genomic potential and do not directly infer in vivo activity or clinical outcomes, taken together they support the view of Gardnerella as an ecologically diverse genus composed of lineages with distinct metabolic capacities, host-interaction mechanisms, and mobile genetic repertoires.

The text now states (lines 397-400):

Although these interpretations are based on comparative genomic potential and do not directly imply in vivo activity or clinical outcomes, taken together they support the view of Gardnerella as an ecologically diverse genus composed of lineages with distinct metabolic capacities, host-interaction mechanisms, and mobile genetic repertoires.

Comment #2:

Regarding Figure 1B. While I appreciate the effort to clarify the relationships between the Piot Biotypes and the cpn60 subgroup/Ahmed clade terminology, it remains true that these relationships are not clear. The number of isolates for which both Piot and sequence-based classifications are available is relatively small and (not surprisingly given they measure different things) they are not concordant. I'm not sure what "illustrative rather than deterministic" means, but the best solution might be to simply remove the arrows and say that there is insufficient data to define these relationships.

Response :

We agree with the reviewer that removing the arrows would increase the accuracy of the figure. We have therefore updated Figure 1 to remove arrows between the Piot *et al*. Biotypes and the Ahmed *et al*./Jayaprakash *et al*. Clades/subgroups and have updated the Figure legend to clarify that arrows are used when there is sufficient evidence to link groups.

Original Figure 1:

REDACTED

Updated Figure 1:

REDACTED

The caption for Figure 1 originally read:

> *Arrows indicate consistent lineages between studies without implying a definitive link.*

The caption for Figure 1 now reads:

> *Arrows indicate consistent lineages between studies where enough evidence exists to link the groups.*

<u>Comment #3</u>

Regarding abbreviations. Explaining the use of these within this paper is fine, but I recommend not suggesting any use beyond this paper. It is always better to spell out organism names and with the exception of figure labels on occasion it is not necessary to abbreviate.

<u>Response</u> :

We thank the reviewer for this comment. While we appreciate the general principle of spelling out species names, we respectfully maintain our position that proposing standardized abbreviations for *Gardnerella* species serves an important purpose beyond this manuscript alone.

The *Gardnerella* field has undergone significant taxonomic expansion in recent years, and the nomenclature to accurately categorize all of these lineages is now extensive. Although we agree with the reviewer that full species names should be used, when possible, we recognize that the use of abbreviations may be useful for other groups within their own scientific publications and figures. The purpose of proposing a consistent abbreviation scheme in this work is to avoid any future inconsistency or confusion with how other groups may refer to these lineages in their manuscripts and figures. By proposing a clear framework here, we provide a foundation that future groups can build upon when naming and referring to any additional novel lineages, thereby promoting clarity and consistency across the field.

That said, we have taken the reviewer's concerns seriously and in order to address them we have 1) revised all figures to include full species names, and 2) edited the text to encourage the use of full species names when possible. We hope this revision strikes an acceptable balance.

The original text read:

To minimize ambiguity in referring to Gardnerella species in this paper, we intentionally propose species names that can be abbreviated with unique two letter abbreviations (Gb, Gg, Gh, Gi, Gk, Gl, Gm, Gp, Gr, Gv and Gw), while individual subspecies were designated with discernable three-letter abbreviations (except for Gardnerella piotii subsp. pickettii and Gardnerella piotii subsp. piotii, which each require four letters, Gppk and Gppt)

The updated text on lines 142-149 now reads:

While we encourage the use of full species names, when possible, we also recognize that abbreviations may be useful within scientific publications and figures. To minimize ambiguity in referring to Gardnerella species in this paper, we therefore intentionally propose species names that can be abbreviated with unique two letter abbreviations (Gb, Gg, Gh, Gi, Gk, Gl, Gm, Gp, Gr, Gv and Gw), while individual subspecies were designated with discernable three-letter abbreviations (except for Gardnerella piotii subsp. pickettii and Gardnerella piotii subsp. piotii, which each require four letters, Gppk and Gppt).

Reviewer 4

Comment #1

I co-reviewed this manuscript with one of the reviewers who provided the listed reports. This is part of the Nature Communications initiative to facilitate training in peer review and to provide appropriate recognition for Early Career Researchers who co-review manuscripts.

Response :

We thank the reviewer and co-reviewer for their careful evaluation of our manuscript and for their constructive feedback throughout the review process. We also appreciate Nature Communications' initiative supporting peer review training. The comments and suggestions provided were valuable in strengthening the manuscript.
